# Intron retention enhances gene regulatory complexity in vertebrates

**DOI:** 10.1186/s13059-017-1339-3

**Published:** 2017-11-16

**Authors:** Ulf Schmitz, Natalia Pinello, Fangzhi Jia, Sultan Alasmari, William Ritchie, Maria-Cristina Keightley, Shaniko Shini, Graham J. Lieschke, Justin J-L Wong, John E. J. Rasko

**Affiliations:** 1 Gene & Stem Cell Therapy Program, Centenary Institute, University of Sydney, Camperdown, 2050 NSW Australia; 2 Sydney Medical School, University of Sydney, Camperdown, 2050 NSW Australia; 3 Gene Regulation in Cancer Laboratory, Centenary Institute, University of Sydney, Camperdown, 2050 NSW Australia; 4 Australian Regenerative Medicine Institute, Monash University, Clayton, 3800 VIC Australia; 5 CNRS, UMR 5203, Montpellier, 34094 France; 6 School of Biomedical Sciences, The University of Queensland, Brisbane, QLD 4072 Australia; 7 Cell and Molecular Therapies, Royal Prince Alfred Hospital, Camperdown, 2050 NSW Australia; 8 Locked Bag 6, Newtown, NSW 2042 Australia

**Keywords:** Transcriptomic complexity, Granulocytes, Evolution, Alternative splicing, Intron retention, Gene regulation

## Abstract

**Background:**

While intron retention (IR) is now widely accepted as an important mechanism of mammalian gene expression control, it remains the least studied form of alternative splicing. To delineate conserved features of IR, we performed an exhaustive phylogenetic analysis in a highly purified and functionally defined cell type comprising neutrophilic granulocytes from five vertebrate species spanning 430 million years of evolution.

**Results:**

Our RNA-sequencing-based analysis suggests that IR increases gene regulatory complexity, which is indicated by a strong anti-correlation between the number of genes affected by IR and the number of protein-coding genes in the genome of individual species. Our results confirm that IR affects many orthologous or functionally related genes in granulocytes. Further analysis uncovers new and unanticipated conserved characteristics of intron-retaining transcripts. We find that intron-retaining genes are transcriptionally co-regulated from bidirectional promoters. Intron-retaining genes have significantly longer 3′ UTR sequences, with a corresponding increase in microRNA binding sites, some of which include highly conserved sequence motifs. This suggests that intron-retaining genes are highly regulated post-transcriptionally.

**Conclusions:**

Our study provides unique insights concerning the role of IR as a robust and evolutionarily conserved mechanism of gene expression regulation. Our findings enhance our understanding of gene regulatory complexity by adding another contributor to evolutionary adaptation.

**Electronic supplementary material:**

The online version of this article (doi:10.1186/s13059-017-1339-3) contains supplementary material, which is available to authorized users.

## Background

Most multi-exon genes (~95%) have more than one alternative splice form due to exon skipping/inclusion, alternative 3′ and 5′ splice site selection, or through intron retention (IR) [1]. Of these modes of alternative splicing, IR is unique as it does typically not contribute to proteomic diversity. IR affects transcripts from up to three-quarters of multi-exonic genes in mammals, yet remains the least understood mode of alternative splicing [2–5].

IR occurs physiologically when the splicing machinery fails to excise introns from primary messenger RNA (mRNA) transcripts leading to the inclusion of premature termination codons (PTCs) in most intron-retaining transcripts [6]. As a consequence, intron-retaining mRNA transcripts are susceptible to degradation via nonsense-mediated decay (NMD) [6, 7]. Thus, IR can reduce gene expression at the post-transcriptional level and thereby imposes an additional level of gene regulation. Indeed, we have shown previously that IR-triggered NMD in differentiating myeloid cells reduces the abundance of cognate RNA and proteins [3]. In this context, several studies have subsequently reported transcripts with included introns detained in the nucleus and not susceptible to NMD [2, 8–12]. Additionally, there are several alternative hypotheses concerning the biological functions of IR [6, 13]. Intron-retaining transcripts may act as sentinel RNAs ready to be spliced and translated on demand, thereby inducing more rapid protein production than de novo transcription and translation [5, 10, 14]. Other downstream effects of IR include the synthesis of novel peptides or protein isoforms, the suppression of protein and/or non-coding RNA (ncRNA) synthesis and the regulation of nuclear mRNA export [2, 3, 15–17]. Normal regulation of IR is essential for physiological cell functions including differentiation capacity and aberrant IR leads to human diseases including cancer [18–21].

Retained introns are known to contain, on average, a higher density of GC nucleotides and are shorter in length compared to non-retained introns [2, 3]. We and others have demonstrated that IR is a conserved mechanism that affects functionally related genes in humans and mice [2, 3, 8]. Although IR has been shown to be conserved across several vertebrate species at a tissue level, such as in the nervous system and brain [2], a thorough analysis of IR in a highly purified and functionally defined cell type has been lacking.

In an evolutionary context, the expansion of alternative splicing has been associated with increased transcriptomic complexity [22]. Although the frequency of alternative splicing reduces with evolutionary distance from primates [23], it is not known whether this is also a characteristic of IR. We aimed to determine whether IR contributes to transcriptomic complexity, to reveal affected biological processes, and to define specific conserved features. To achieve these goals, we analyzed IR in highly purified neutrophilic granulocytes from three mammalian species, one avian and one representative of the teleost fish, i.e. in total, five vertebrate species spanning 430 million years of evolution (Table 1) [24].

**Table 1:** Genomic characteristics of intron-retaining mammalian and vertebrate species

	Genome size (GB)	Chromosomes	pc genes	nc genes	sRNA	lncRNA	Pseudogenes	GC (%)	Introns Mb (%)^a^
Human	3.5	46	20,296	25,173	7703	14,889	14,424	41.3	1512.7 (52.2)
Mouse	3.4	40	22,547	12,583	5530	6489	8770	42.3	992.7 (37.4)
Dog	2.3	78	19,856	3774	3348	426	950	41.3	796.6 (33.3)
Chicken	1.07	78	15,508	1558	1408	150	42	41.9	403.1 (39.0)
Zebrafish	1.46	50	25,642	6008	3172	2741	293	36.7	722.2 (52.7)

Sources of information are indicated in the “Methods” section. Data on introns were determined using the featureBits program of the UCSC genome browser.

^a^Percent of the genome

*pc* protein-coding, *nc* non-coding, *sRNA* small RNA, *lncRNA* long non-coding RNA

Investigation of IR in a cell type with conserved function across diverse species can potentially reveal unique characteristics, which would otherwise be masked when studying mixed populations of cells [2]. Neutrophilic granulocytes offer a discrete, well-defined cell type with phylogenetically conserved functions that serve as an excellent exemplar to study mechanisms of gene expression control. They are the most abundant cells of the innate immune system and consistently exhibit potent anti-microbial defenses since before the evolutionary divergence of teleost fish [25]. Our experimental design provides a tightly controlled model that allowed us to examine the relationship between IR and transcriptomic complexity.

We demonstrate that IR affects many orthologous or functionally related genes and that intron-retaining transcripts have very similar characteristics in all species investigated. For example, we found that intron-retaining genes are transcriptionally regulated from bidirectional promoters. The strong anti-correlation between the number of genes affected by IR and the number of protein-coding genes in the genome of individual species suggests that IR provides a mechanism of enhancing transcriptomic complexity.

It is unknown whether IR acts independently of other post-transcriptional mechanisms of gene expression control. In examining the relationship between IR and microRNA (miRNA)-mediated gene regulation we found that intron-retaining genes have significantly longer 3′ UTR sequences that are enriched for miRNA binding sites. Our results suggest that IR is an evolutionary well-conserved form of alternative splicing that orchestrates post-transcriptional gene expression control.

## Results

### The function of IR is conserved over 430 million years

We have shown previously that IR affects similar biological processes during hematopoietic differentiation in human and mouse [3]. To study the functional conservation of IR events in species spanning 430 million years, we identified IR in terminally differentiated granulocytes using the IRFinder algorithm [26] (see “Methods”). For every intron the algorithm computes the IR ratio (in the range of 0–1), which is an approximation of the proportion of total transcripts that retained the given intron. More specifically, the IR ratio is the ratio of the median read coverage of the intron to that of its flanking exons. We defined a threshold for the IR ratio (*IR_ratio* = 0.1) in order to consider only biologically meaningful cases of IR for further analysis.

**Fig. 1:**
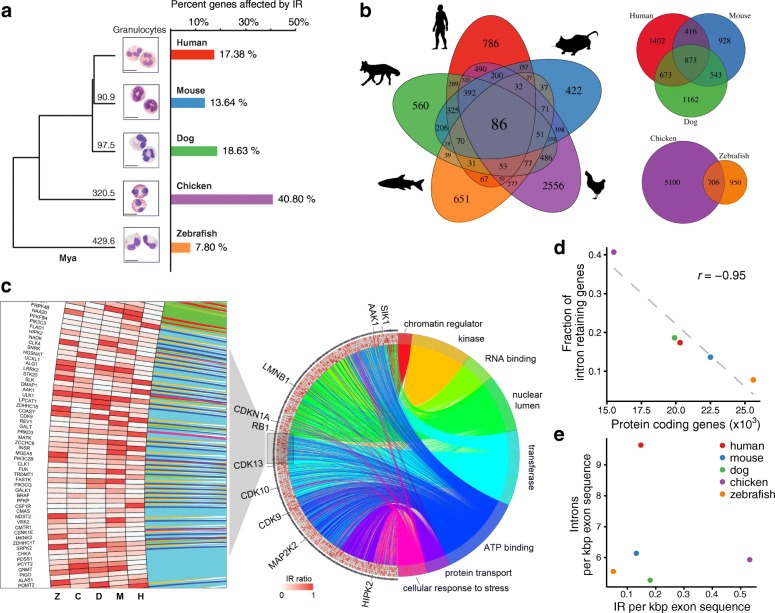
IR conservation in mammalian and vertebrate species. **a** *Phylogenetic tree* of species under investigation and morphology of FACS sorted human, mouse, dog, chicken, and zebrafish granulocytes (Mya = million years ago) following Giemsa or Wright staining. The *horizontal bar plot* shows the fraction of expressed genes affected by IR in each species. **b** The five-way symmetric *Venn diagram* shows the intersections of orthologous intron-retaining genes between species. Eighty-six orthologs are conjointly affected by IR in all five species. The three-way asymmetric *Venn diagram* shows the intersecting gene sets of intron-retaining orthologs in placental mammals (human, mouse, dog), while the asymmetric two-way *Venn diagram* below illustrates the intersection of intron-retaining orthologs in the non-placental vertebrates (chicken and zebrafish). **c** *Circos plot* illustrating links between genes and annotation terms that are repeatedly enriched in the species-specific gene clusters. The right semicircle depicts the enriched terms. The *left semicircle* includes five *concentric rings* that represent color-coded IR ratios of orthologous genes in all five species, starting from human (H), mouse (M), dog (D), chicken (C), and zebrafish (Z). *Left*: A magnified section of the concentric rings. Orthologous genes sometimes do not have consistent IR values across the species; however, the IR functional specificity is conserved by targeting functionally related genes. A scalable version of this figure in vector format is provided in Additional file 5. **d** IR data from granulocytes exhibits a strong anti-correlation (Pearson correlation; r = –0.95) between the fraction of expressed intron-retaining genes and the number of protein-coding genes in a genome. **e** Number of retained introns per kbp exon sequence in relation to the average number of introns per kpb exon sequence in a genome

Of the five species, the representative of the ray-finned fishes (zebrafish) has the lowest fraction of expressed genes that are affected by IR (7.8%). The group of more closely related mammalian species have a similar IR abundance with 13.6% of the expressed genes affected in mouse, 17.4% in human, and 18.6% in dog. The exception in this study is the avian representative, in which a great proportion of expressed genes (40.8%) retained one or more introns in their mRNA transcripts (Fig. 1a). Taken together, in all five species we found a considerable fraction of expressed genes that are affected by IR (see Additional file 1).

Next, we clustered all known orthologous genes based on their IR pattern in the five species under investigation. We found gene clusters in which IR events are exclusive to one particular species. While these species-specific clusters represent distinct sets of genes, annotation enrichment analysis revealed that similar terms are over-represented in association with all of these gene sets, as well as those in which the IR pattern is similar in several or all species (Additional file 2: Figure S1). This suggests that IR is a global control mechanism affecting functionally conserved biological processes independent of specific effector genes. Although we note that mutually enriched terms are rather general, they differ from terms that were found enriched in other studies of IR in purified cells (Additional file 2: Table S1).

Interestingly, functionally enriched classes of genes include phosphoproteins, kinases, and genes involved in alternative splicing, i.e. groups of intron-retaining genes that realize a multitude of gene expression and protein activity control mechanisms. Consistent with this functional enrichment, Theilgaard-Mönch et al. observed an overall decline of proliferative and general cellular activity during terminal granulocytic differentiation [27]. This is plausible given that IR typically induces NMD and thereby negatively affects widespread mRNA and protein expression [3, 28]. Analogous to these observations is a recent finding that shows decreasing IR ratios associated with increasing levels of fully spliced mRNAs during T cell activation, to facilitate a prompt cellular response to extracellular stimuli [5].

In all species there are large numbers of intron-retaining genes with functions that are relevant to organelle lumina, most commonly the nuclear lumen. This result confirms our previous observations concerning the importance of IR in the control of granulocyte nuclear morphology [3]. The organelle lumina group includes genes that we also identified in our previous study in mouse and human granulocytes [3], e.g. *Ddx5* (all species), *Ddx3x*, *Lbr*, *Atf4*, *Hspa5*, *Ing4* (human, mouse, dog, chicken), *Dnmt3a* (mouse, dog, chicken), *Hnrnpa2b1* (human, dog), *Lmnb2* (mouse, chicken), and *Lmnb1* (mouse).

Other well-conserved classes of intron-retaining gene orthologs include: kinases (e.g. *Cdkn1a*, *Cdk9/10/13*, *Map2k2*, *Sik1*), RNA-binding proteins (e.g. *Upf1*, *Dhx58*, *Ddx17*, *Upf3b*); ATP binding proteins (e.g. *Ddx5*, *Ddx3x*, *Hspa5*, *Eif4a1*, *Ddx39*, *Atp2a3*, *Adrbk1*); and protein transporters (*Hspa5*, *Hsp90aa1*, *Hsp90ab1*) (see Additional file 3).

We conclude that although specific intron-retaining genes may vary between species, they are conserved in functional clusters. Our results suggest that IR is a function-centric rather than gene-centric mechanism of coordinating gene expression (Fig. 1c). Nonetheless, there are surprisingly large numbers of gene orthologs (n = 86) in which IR-mediated gene regulation is conserved in the granulocytes of all five species under investigation, i.e. species that shared a common ancestral genome sequence 430 million years ago (Fig. 1b). The number of conserved intron-retaining genes is even tenfold higher among the placental mammalian species (human, mouse, dog), which share as many as 873 intron-retaining gene orthologs. The non-mammalian vertebrates share 706 intron-retaining gene orthologs (Fig. 1b). We also analyzed conservation of IR on a per-intron basis by determining orthologs of retained introns in the other species. The results suggest that although IR on a per-intron basis is less conserved, conservation is still remarkable among the mammalian species (Additional file 2: Figure S2b).

In summary, our data indicate that IR is a well-conserved mechanism of process- or function-centric gene regulation in mammalian and vertebrate species, affecting a large number of orthologous and functionally related genes.

### IR preserves functional complexity in species with fewer genes

It has previously been shown that the frequency of alternative splicing events reduces with evolutionary distance from primates [23]. However, our observations of IR events in the investigated vertebrates contradict this observed trend (Fig. 1a). To determine whether retained introns, acting as gene expression control elements, preserve complexity in vertebrate species, we compared the fractions of intron-retaining genes in each species with the number of protein-coding genes in their genome. A strong anti-correlation exists between these two variables (*r* = –0.95, Pearson correlation; Fig. 1c). In contrast to other forms of alternative splicing that introduce proteomic complexity to the cell [29–32], our data indicate that IR introduces transcriptomic complexity in species with lower numbers of protein-coding genes. The most extreme example in our study is chicken in which > 40% of the 8911 expressed genes (fragments per kilobase of transcript per million mapped reads [FPKM] ≥ 1) in granulocytes possess retained introns, the largest fraction in all species investigated. Chicken has by far the smallest number of protein-coding genes among the five species, with a total of 15,508. On the other end of the spectrum is zebrafish, in which 7.8% of the 768 expressed genes contain retained introns, which is a relatively small fraction in a genome that has evolved complexity via a large number of protein-coding genes (n = 25,642). While IR anti-correlates with the number of protein-coding genes in a genome, the correlation does not hold when we instead compare IR to the number of expressed genes. Our observations indicate transcriptional control is the dominant mechanism of gene expression control in cases where few genes are expressed (zebrafish). Post-transcriptional control of gene expression including IR is more dominant in cases where many genes are expressed (chicken). In order to reinforce this thesis, we extracted the number of transcriptional regulators (transcription factors, transcription co-factors, and chromatin remodeling factors) for each species from the AnimalTFDB database [33] and found that this number anti-correlates (*r* = –0.73, Pearson correlation) with the number of expressed genes in our samples. In zebrafish, where there are abundant transcriptional regulators, the number of expressed genes is low. We argue therefore in zebrafish that IR is not as important comparatively as it is in organisms like avian species where IR is a dominant mechanism of post-transcriptional gene expression control. Of note, intra-sample comparisons of gene expression values resulted in no consistent picture, showing lower median expression levels (FPKM values) of intron-retaining genes only in dog, chicken, and zebrafish (Additional file 2: Figure S3).

Despite the number of intron-retaining genes, it is also important to take into account the density of retained introns. Eukaryotic species differ substantially in their intronic density [34]. Some species have less than 100 introns in their total genome but through spliceosomal intron evolution higher developed organisms have, on average, up to eight introns per gene [35]. In our present study humans exhibit the highest density of introns with 9.6 introns per kilobase-pair (kbp) of exonic sequences. However, chicken has the highest density of retained introns (0.53 retained introns/kbp exon), which is almost three times more than the species with the second highest density (dog, 0.18 retained introns/kbp exon; Fig. 1e).

Our observations suggest that IR is a phenomenon, among others, implemented to preserve transcriptomic complexity in genomes with fewer protein-coding genes. Here, complexity is effected by realizing fine-tuning of gene expression control and thereby allowing a cell to adapt to environmental changes [5]. Therefore, we propose that IR enhances gene regulatory complexity in vertebrate species.

### Characteristics of retained introns are well-conserved

**Fig. 2:**
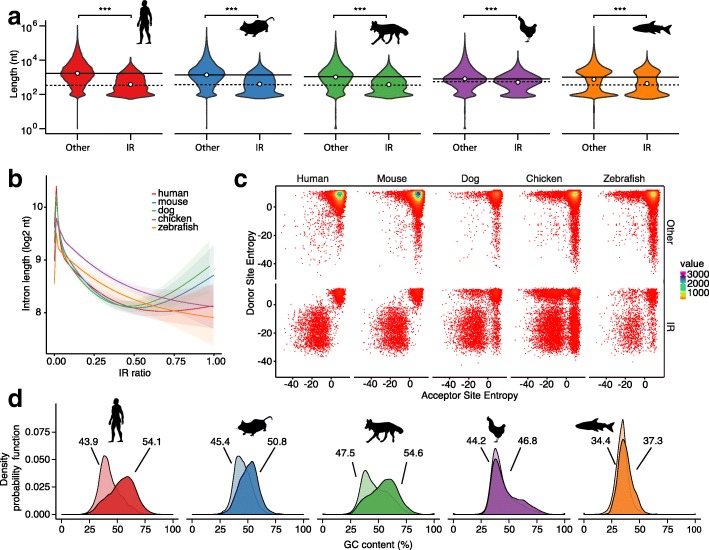
Characteristics of retained introns. **a** *Violin plots* showing the log10 length distribution of non-retained (*left violin* in each subplot) and retained introns (*right violin*). Mann–Whitney U test was used to determine significance, denoted by *** (*p* < 0.001). **b** Generalized additive model with smoothness estimation of the intron length/IR ratio relationship. **c** Bivariate *histograms* illustrating strengths of splice site pairs (as maximum entropy) [37] of retained introns and all other introns using hexagon binning (100 × 100 bins). **d** Density of the GC content in retained (*dark color*) and non-retained introns (*light color*). Numbers indicate the mean GC content

To define features that are conserved we compared characteristics of retained introns and their host genes in the investigated species. First, we compared the length of retained introns in granulocytes and found very similar distributions in human, mouse, dog, and chicken, where the average length of retained introns is consistently shorter than that of non-retained introns (Fig. 2a, Additional file 2: Table S2 and Figure S4). Interestingly, as the genome-specific intron length (*μ*) increases with genome size (*μ*_*Chicken*_ = 797 nt; *μ*_*Zebrafish*_ = 1023 nt; *μ*_*Dog*_ = 1091 nt; *μ*_*Mouse*_ = 1402 nt; *μ*_*Human*_ = 1677 nt) so does the fold difference (*FD*) between the length of non-retained introns and retained introns (*FD*_*Chicken*_ = 1.4; *FD*_*Zebrafish*_ = 2.8; *FD*_*Dog*_ = 3.1; *FD*_*Mouse*_ = 3.7; *FD*_*Human*_ = 4.9). The length of retained introns also decreases with higher IR ratios in chicken and zebrafish; however, in the mammalian species the negative trend is reversed at an IR ratio of about 0.5 in mouse and dog, and at an IR ratio of ~ 0.7 in human (Fig. 2b). Nevertheless, the majority (more than 93%) of retained introns examined have an IR ratio < 0.5 (Additional file 2: Figure S5).

Others have shown previously that weak splice sites favor IR [36]. Using maximum entropy modeling [37], we confirmed this characteristic of retained introns in our data and thus demonstrate that this feature is well-conserved in all species investigated (Fig. 2c). The pattern of splice site pair entropies shown in the bivariate histograms suggests that often both splice sites of retained introns are weak, thereby predisposing them for retention.

Retained introns are known to have, on average, a higher GC content compared to non-retained introns [2, 3]. We confirmed this trend in this wider spectrum of species, where the highest GC content was found in retained introns of the mammalian representatives (Fig. 2d). This reinforces our previous observations in human and mouse granulocytes as well as in murine megakaryocytes and erythrocytes [3, 4].

Intron-flanking domains expanded faster during proteomic evolution than other protein domains [38]. These mobile domains have a strong preference for phase 1 introns. Although retained introns also exhibit a phase 0 excess as observed in all introns [39], our data indicate a slight but consistent shift of the intron phase distribution away from phase 0 in retained introns in all five species (Additional file 2: Figure S6).

We have shown previously that many retained introns harbor PTCs [3]. By comparing the density of PTCs in retained vs non-retained introns, we found that retained introns incorporate slightly but significantly lower PTC densities (Additional file 2: Figure S7). The underrepresentation of PTCs in retained introns might be due to the contribution of intron-retaining isoforms that are not destined for NMD.

**Fig. 3:**
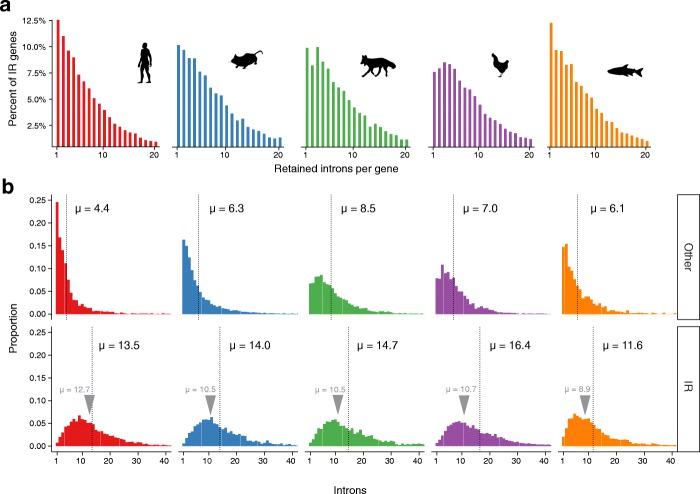
Characteristics of intron-retaining genes. **a** Histograms of the number of retained introns in intron-retaining genes. **b** Distribution of intron number in transcripts without (*upper panel*) and with IR (*lower panel*) as a proportion of all transcripts. Genes that do not contain retained introns (Other) include expressed genes (FPKM > 1) only. *Gray arrows* above the *curves* indicate the average number of introns per gene in each species

Intron-retaining transcripts in granulocytes are often subject to degradation via NMD mostly triggered by PTCs that facilitate detection by UPF1 triggering NMD by interacting with UPF2 and UPF3 bound to the next exon-junction complex [3, 7]. Although conditions exist in which a PTC does not lead to NMD [40], for the purpose of gene regulation via IR no more than one retained PTC-containing intron should be required. Surprisingly, in intron-retaining genes the number of retained introns is proportional to the total number of introns in a gene (Additional file 2: Figure S8). It needs to be noted, however, that the relatively short read length (201 bp) in Illumina RNA sequencing (RNA-seq) does not allow conclusions about the number of introns that are retained in a single transcript. Both scenarios are therefore possible: (1) different transcripts have distinct individual introns retained; or (2) transcripts harbor several retained introns. In most of the cases in human, mouse, and zebrafish, it is only one intron that is retained with a steady decrease in the frequency of cases where two or more different introns are retained (Fig. 3a). In dog, there are as many genes with three different retained introns as there are cases where only one intron is retained. Notable in this context is once again chicken, where in most of the cases three or four introns per gene are retained.

We also observed that transcripts with a larger number of introns are more prone to be affected by IR (Fig. 3b). This observation is consistent with previous studies that have identified the increase of the number and length of introns in higher developed organisms as a way to induce genomic complexity [41, 42].

**Fig. 4:**
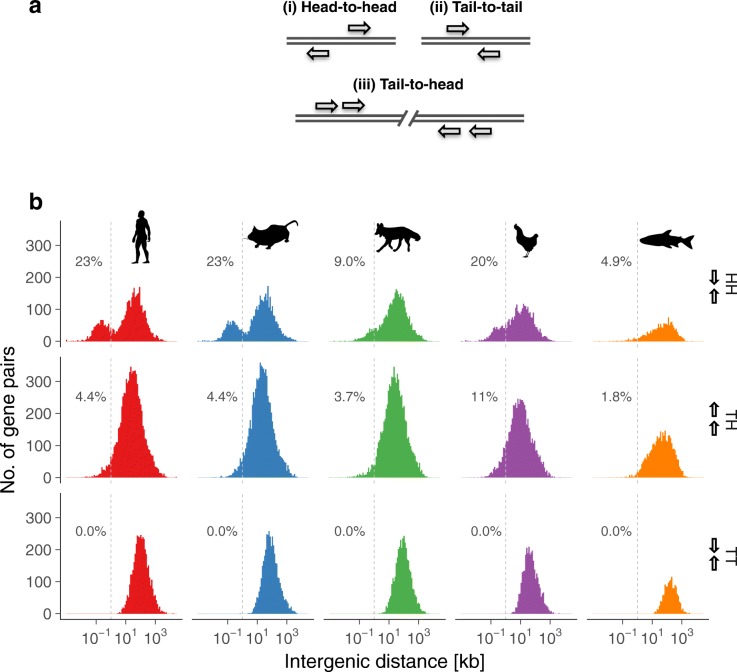
Bidirectional promoters in intron-retaining genes. **a** Gene orientation scheme with arrow heads at the 3′ end. **b** *Histograms* of binned intergenic distances between intron-retaining genes (*right*). The intergenic distance is determined as distance (in kb) between the transcription start sites of two genes (–/+; HH) or end of transcripts (+/–; TT), when on opposite strands, and between end of transcript and transcription start site, when both genes are on the same strand (+/+ or –/–; TH). The percentages indicated in each plot refer to the fraction of gene pairs with an intergenic distance of ≤ 1 kb

Bidirectional gene pairs are co-transcribed due to a mutual promoter region. It was shown previously that among adjacent human housekeeping genes there is enrichment in bidirectional gene pairs that reside in close proximity (<1 kb nt distance) [43–45]. To explore possible post-transcriptional repression via IR, we measured the distances between gene pairs that involve two intron-retaining genes. Furthermore, we classified gene pairs in accordance to the published system as depicted (Fig. 4a) [46]. We consider head-to-head gene pairs as bidirectional and gene pairs with a distance < 1 kb as putatively co-regulated. It has been shown that many gene pairs in human and mouse share a mutual promoter region [46]. We have confirmed these findings (Additional file 2: Figure S10); however, the fraction of the gene pairs is smaller than that in the group of genes with retained introns compared to non-retained (human: 16% vs 23%; mouse: 14% vs 23%). We found that the fraction of gene pairs sharing a mutual promoter is much larger in the group of genes with retained introns (human: 23%; mouse: 23%; Fig. 4b) compared to genes without retained introns (human: 6.4%; mouse: 3.5%; Additional file 2: Figure S10). Strikingly, frequencies of gene distances show an enrichment of bidirectional promoters also in dog and chicken (Fig. 4b, top row). The observed enrichment of bidirectional promoters could be an indication of post-transcriptionally dominated gene regulation in species/cells with a large number of expressed genes. This would explain why no enrichment has been observed in zebrafish with the lowest number of expressed genes and the largest number of transcriptional regulators in this cohort. Taken together, common regulation of transcriptional initiation or other processes occurring at the promoter may play a role in intron-retaining genes and is well-conserved.

In examining diverse species in a well-defined cell, our study corroborates previously observed characteristics of retained introns (Additional file 2: Table S3). Additionally, we demonstrated that these characteristics are well-conserved. Patterns of IR at the gene level suggest that often several different introns are retained and that transcripts with a larger number of introns have a higher probability of IR events (Additional file 2: Figure S11). These observations can be linked to previous hypotheses that the ratio of non-coding to protein-coding DNA rises as a function of transcriptomic complexity [47] and that introns fulfill many essential functions, including the regulation of gene expression, in intron-rich species [48].

### Intron retention complements miRNA-mediated gene regulation

**Fig. 5:**
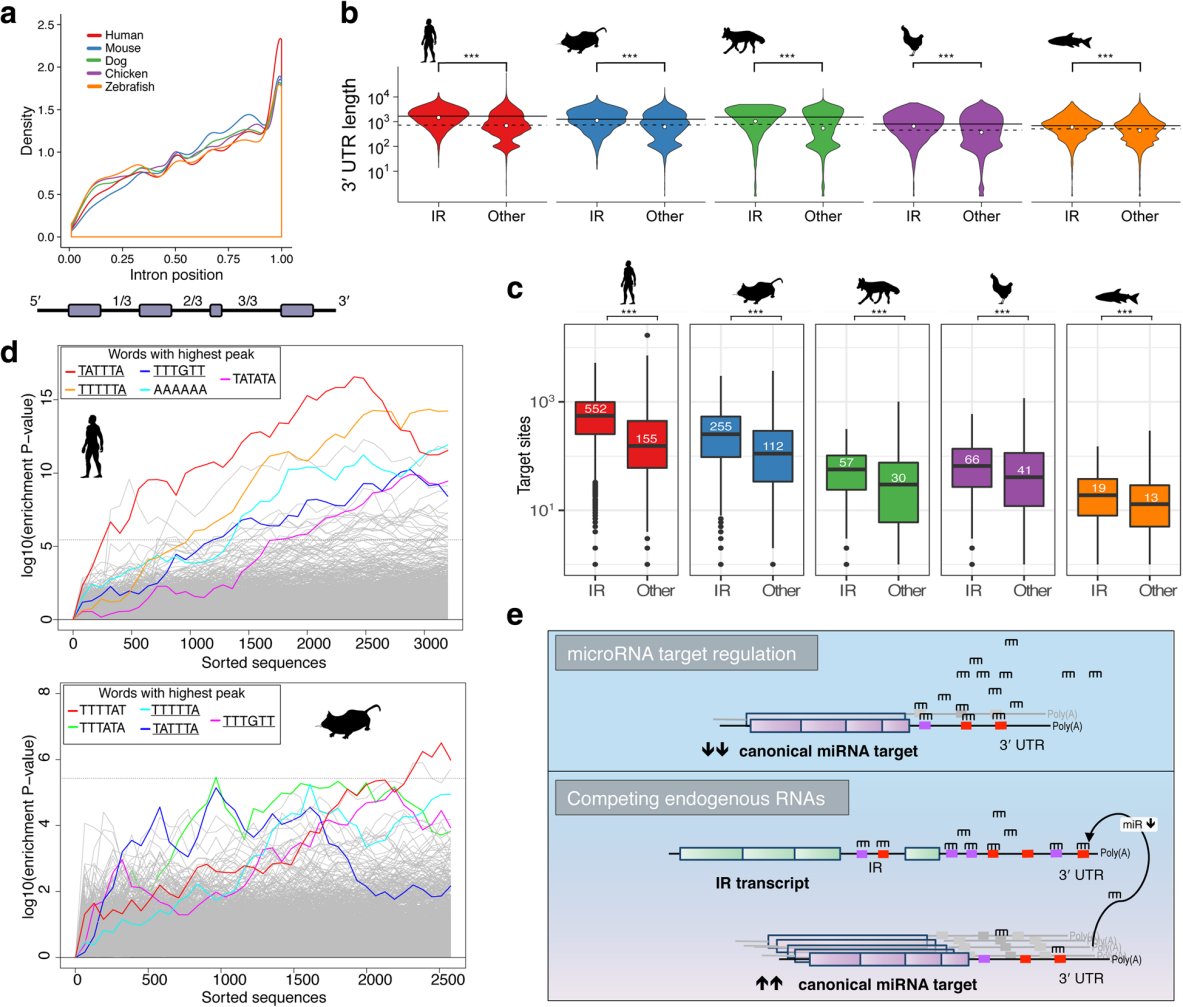
Relative position of retained introns and miRNA binding site enrichment. **a** Probability density function of the position of retained introns in relation to the other introns in the gene structure. Values between 0 and 1 represent the relative intron position, which is calculated by dividing the intron position by the total number of introns in a transcript. **b** Densities of 3′ UTR lengths as *violin plots*. Densities of 3′ UTR sequence lengths in transcripts with (IR) and without retained introns (Other). The *solid* and *dashed horizontal lines* mark the median 3′ UTR length of genes with and without retained introns, respectively, and the *white dots* their mean. Genes that do not contain retained introns (Other) include lowly and non-expressed genes. **c** Comparison of the number of predicted miRNA binding sites in the 3′ UTR sequences of genes with retained introns and non-intron-retaining genes. The white numbers indicate the median value, illustrated also by a *horizontal line* in each box. Genes that do not contain retained introns (Other) include lowly and non-expressed genes. **d** Sylamer [55] *plots* illustrating 6mer seed sites enriched in the 3′ UTR sequences (*x*-axis) of intron-retaining genes in human and mouse based on a hypergeometric significance test. The canonical polyadenylation signal (AATAAA), which is also enriched in both species, is not highlighted. Mutually enriched seed site sequences are *underlined*. The *horizontal dotted line* represents an E-value threshold (Bonferroni-corrected) of 0.01. The corresponding plots for dog, chicken, and zebrafish are in Additional file 2: Figure S17. **e** Model of intron-retaining transcripts as competing endogenous RNAs. Wilcoxon test was used to determine significance, denoted by ^***^ (*p* < 0.001)

Independent observations show that retained introns predominantly reside near the 3′ end of a gene transcript [2, 3, 18]. We further determined the genomic distribution of retained introns to confirm previous findings and demonstrate the strong conservation of this 3′ prevalence in IR (Fig. 5a). Although this pattern is consistent, in case of very long retained introns, the 3′ prevalence is only preserved in zebrafish (see Additional file 2: Figure S9). Our observations with respect to the length and location of retained introns correspond with the trend of a decrease in intron length towards the 3′ ends of genes in vertebrate genomes [49]. This also provides reassurance that the observed 3′ prevalence of IR is neither caused by a technical nor analytical artifact. We performed gene body coverage analysis and did not find an overall bias in the mapping of any regions of genes. Thus, the 3′ prevalence of IR is not due to a technical bias (Additional file 2: Figure S9).

Interestingly, in human and mouse many retained introns are located in the 3′ UTR (Additional file 2: Figure S12), i.e. they reside between untranslated exons and are typically spliced out during the mRNA maturation process [50]. This observation suggests the existence of novel yet unannotated 3′ UTR isoforms. Because longer 3′ UTR sequences may harbor additional miRNA binding sites [51], we wondered whether intron-retaining transcripts might act as competing endogenous RNAs or miRNA sponges [6, 52]. To further investigate this possibility, we compared the 3′ UTR lengths of genes with and without retained introns in their cognate mature transcripts. Surprisingly, we found that the mean 3′ UTR length of intron-retaining genes is significantly longer than that of non-intron-retaining genes in all species (Fig. 5b). The difference increases in species that are evolutionarily closer to human, while in humans the median 3′ UTR sequence of intron retaining genes is more than twice as long compared to the median in non-intron-retaining genes (Fig. 5b). Our evidence suggests that IR preferentially occurs in longer, more complex genes.

We predicted miRNA binding sites in the 3′ UTR sequences of genes with retained introns and all other genes (not incorporating any 3′ UTR intron sequences). Surprisingly, in all species the number of putative miRNA binding sites is drastically increased in genes with retained introns (Fig. 5c). The difference in miRNA binding sites in intron-retaining genes relative to non-intron-retaining genes increases with species that are evolutionarily closer to human (zebrafish: 46%; chicken: 61%; dog: 90%; mouse: 126%; and human: 256% increase). With retained introns in the 3′ UTR, the number of predicted miRNA binding sites increases further by 186.3, 124.1, 18.1, 70.4, and 28.9 additional sites, on average, in human, mouse, dog, chicken, and zebrafish, respectively. This leads us to hypothesize that the expression of genes subject to IR is also controlled by miRNAs. MiRNA target prediction also revealed a significantly higher density of putative miRNA binding sites in sequences of retained introns compared to non-retained introns (Additional file 2: Figure S13). IR transcripts could therefore function as miRNA sponges to indirectly regulate other transcripts by modulating the available pool of miRNAs (Fig. 5e). Six examples in which retained introns may facilitate a miRNA sponge effect were derived from our previous data comparing promyelocyte against granulocyte transcriptomes in mice (Additional file 2: Figure S14; GEO accession numbers: GSE48307 [53], GSE57624 [54]). Moreover, we show based on public Ago2 HITS-CLIP data that Argonaute and possibly associated miRNAs can potentially bind to the predicted miRNA binding sites in introns (Additional file 2: Figure S15). We have also illustrated the putative effects of different miRNA expression and intron-retaining transcript levels on the expression of endogenous miRNA targets using a kinetic model based on ordinary differential equations (Additional file 2: Figure S16).

In addition, we identified significantly over-represented sequence motifs (putative miRNA seed sites) in the 3′ UTR sequences of intron-retaining genes using Sylamer [55]. Interestingly, we found strong similarities among the enriched sequence motifs in mammalian species (Fig. 5d; Additional file 2: Figure S17 and Table S4) and the motifs are, on average, more conserved than their flanking regions (Additional file 2: Figure S18). Taken together, these results indicate that IR is a mechanism of post-transcriptional gene regulation that complements miRNA-mediated target repression. However, given that many 3′ UTR introns are retained and that intron-retaining transcripts have more miRNA binding sites, they may also act as miRNA sponges as we previously proposed [6] (Fig. 5e). In support of this notion is our observation that the enriched sequence motifs are also over-represented in the 3′ UTRs of genes upregulated in hematopoietic differentiation, putatively benefiting a relief from miRNA-induced expression control through intron-retaining miRNA sponge genes (Additional file 2: Figure S20).

## Discussion

Of all forms of alternative splicing, least is known about IR. One reason is that IR events are difficult to detect because cellular surveillance mechanisms like NMD can rapidly degrade transcripts with retained introns, although under certain circumstances primary transcripts can evade NMD by detention in the nucleus [2, 8, 9, 11, 12]. Such differences can be separated experimentally by performing nuclear:cytoplasmic fractionation, as we have previously described [3]. Furthermore, read mapping is challenging as introns are long and abundant in low-complexity regions [56]. Therefore, most transcriptomic studies focusing on alternative splicing have overlooked this type of regulation, despite its potential impact and the multitude of possible downstream effects [6].

In this in-depth phylogenetic exploration of IR in three mammalian and two non-mammalian vertebrate species, which shared a common ancestral genome 430 million years ago, we have shown that IR provides a conserved and orchestrated mechanism of post-transcriptional gene regulation. Since different subtypes of cells have vastly different mRNA splicing patterns associated with different functions, in this study we investigated IR in a highly purified and functionally defined cell type. By adopting this meticulous approach to benchmarking IR, we have extracted unique insights regarding the conservation and function of IR across species, which would otherwise likely be obscured when performing whole-tissue analysis [2, 3, 57, 58].

A surprising outcome arising from this study was the strong anti-correlation between the number of intron-retaining genes and the number of protein-coding genes in a genome (Fig. 1d). This contrasts with the known increase in intron quantity with genome size, thereby facilitating increased transcriptomic complexity [34]. The false assumption that developmental complexity would be reflected by the number of protein-coding genes in a genome, referred to as the *G-value paradox* [59], was partially explained by alternative splicing phenomena that introduce proteomic complexity through novel protein isoforms [22, 29–31, 42]. An alternative measure suggested to account for genomic complexity is the intron density in eukaryotic organisms [30], which relates to the capacity in realizing alternative splicing events [5, 34]. By studying IR in detail, we have observed how alternative splicing utilizes introns as *cis*-acting regulatory gene elements to post-transcriptionally fine-tune gene expression. Therefore, we suggest that IR increases gene regulatory complexity and refer to: (1) the increased number of mRNA isoforms detectable due to IR; (2) the increased sophistication in gene expression fine-tuning (possible benefits illustrated in Additional file 2: Figure S19a/b) [60]; and (3) IR-induced complexity on a molecular network level (i.e. gene regulatory networks, metabolic networks, signaling networks) by introducing dose-dependent non-linear dynamics (Additional file 2: Figure S19b/c) [61]. However, the ultimate function of IR can only be evaluated when the fates of intron-retaining transcripts are determined [56]. Therefore, we cannot rule out other explanations for the observed anti-correlation between the number of intron-retaining genes and the number of protein-coding genes in a genome. For example, the relative absence of IR transcripts in zebrafish granulocytes, which also express a comparatively small number of genes, could either indicate an absence of IR, or it could reflect more efficient degradation of IR transcripts than in chicken with both a high number of expressed genes and high incidence of detected IR.

Another important observation from this analysis is that intron-retaining genes harbor a larger number of miRNA binding sites (Fig. 5c), mainly generated by the presence of longer 3′ UTR sequences than occur in other genes (Fig. 5b). It has been shown that the length of 3′ UTRs is correlated with morphological complexity in metazoan species [62] and that gene regulation by multiple and cooperating miRNAs mediates enhanced target repression [63–65]. This indicates that IR-mediated decay and miRNA-induced translational repression may be complementary mechanisms orchestrating post-transcriptional gene expression control.

Our analysis reveals that IR does not just affect many gene orthologs but also encompasses other functionally related genes, suggesting that IR is a function-centric form of gene regulation. Many of the intron-retaining genes are downregulated in differentiated granulocytes. We found, for example, 674 human intron-retaining genes to be more than twofold downregulated in the study of Theilgaard-Mönch et al., in which the authors compare gene expression profiles of human promyelocytes, myelocytes, and neutrophils [27]. The authors describe a general decline of proliferative and general cellular activity during terminal granulocytic differentiation. Here, IR seems to be a crucial regulatory factor. Downregulated genes that show IR are enriched in acetylation-related genes and splicing factors, as well as phosphoproteins and kinases and thus affect general gene expression and activity on the transcriptional, post-transcriptional, and post-translational levels, respectively (Additional file 2: Figure S21). This may also explain the G0/G1 arrest and downregulation of kinase expression observed in most end-stage differentiated neutrophil granulocytes in the study by Klausen et al. [66].

The exact mechanisms leading to IR remain to be elucidated; however, it is known that certain features increase the likelihood of an intron to be retained [36, 67]. These features are mainly composed of *cis*-regulatory elements marking characteristics of retained introns and their host genes [68, 69]. In our phylogenetic study of IR in five species we confirmed conservation of such features including weaker splice sites, a higher GC content, and a shorter length of retained introns. Furthermore, we found that retained introns are predominantly located near the 3′ termini of transcripts and have lower PTC densities than non-retained introns.

Most of the observations that we found conserved in all species are particularly pronounced in the avian representative. Chicken granulocytes contained the most intron-retaining genes both in number and in relation to the total number of expressed genes (~40%). Chicken also has the highest density of retained introns; however, their median length is larger than that observed in the other species (Fig. 2a). This is surprising because intron size correlates with genome size and bird introns are typically shorter than that of mammals [49]. This apparent paradox may have two explanations: (1) retained introns in chicken have other functions beyond regulating the expression of their host genes; or (2) they may contain more *cis*-regulatory elements that interact with splicing factors. Moreover, many of the chicken genes are presumably co-regulated (15% of genes and 20% of the intron-retaining genes share a mutual promoter region) and thus are more dependent on post-transcriptional gene regulation (Fig. 4, Additional file 2: Figure S10). This observation contradicts an earlier statement made by Koyanag et al. who studied the evolution of bidirectional gene pairs that share a mutual promoter region and found that enrichment in bidirectional gene pairs is only detectable in mammals and not in other eukaryotes [46].

However, the variation in IR abundance may also be explained by cell biological nuances in each species. Chicken heterophils for example are exceptional in not having a myeloperoxidase-like activity whereas this is the most abundant protein in neutrophils of the other species [70]. In general, IR calling is influenced by sequencing depth and annotation quality, i.e. the more reads and annotated genes/introns the more IR events should be detected. However, this has a negligible impact on our analysis, as the sequencing depth is comparable in all experiments. The fact that the number of retained introns detected in chicken exceeds that of all other species (including human, with the highest number of annotated introns) suggests that our results and conclusions are not biased by annotation quality.

## Conclusions

In summary, our study has provided a definitive documentation of the conserved characteristics exhibited by IR in vertebrate granulocytes, including humans. We have provided new insights that support the notion of IR as an independent mechanism of gene regulation that may interfere with or complement other forms of post-transcriptional gene regulation. In IR we see a form of alternative splicing that realizes a feature contributing to gene regulatory complexity thereby facilitating organismal propensity for adaptation.

## Methods

### Primary granulocytes

A whole blood sample was obtained from a healthy male individual. Peripheral blood leukocytes were separated from red cells and platelets using dextran sedimentation and Ficoll density separation. Human granulocytes (CD11b + CD15+) were isolated using fluorescence-activated cell sorting (FACS) as previously described [3].

Primary mouse granulocytes from bone marrow of male C57BL/6J mice (8–10 weeks) were purified using FACS as previously published [3, 71].

Peripheral blood from ten male dogs (Beagles aged 1–8 years) were collected from Novartis Animal Health. Dog granulocytes were purified using Percoll density separation as previously described [72], followed by FACS using a monoclonal antibody against canine neutrophils (CAD048A, Monoclonal Antibody Center, Washington State University). Purity of granulocytes was > 95% based on morphological assessment.

Chicken granulocytes were isolated from peripheral blood of six male Ross breed chickens (*Gallus gallus domesticus*) aged eight weeks. Following blood collection, heterophil-granulocytes were isolated as previously described [73]. Briefly, whole blood was diluted 1:1 with RPMI 1640 media (Sigma) containing 1% methylcellulose (25 centipoises; Sigma) and centrifuged (25 g, 30 min at 4 °C). The supernatant was transferred to a new tube, washed with calcium and magnesium-free Hank’s balanced salt solution (HBSS, 1:1; Sigma), and layered onto discontinuous Ficoll-Histopaque (Sigma) gradient (specific gravity 1.077 over specific gravity 1.119). The gradients were centrifuged at 250 g for 60 min at 4 °C. After centrifugation, the Histopaque layer containing the granulocytes was collected at the second interface 1.077/1.119 and transferred to a new Falcon tube. Cells were washed three times in RPMI 1640 media and pelleted by centrifugation at 4 °C, 10 min, 200 g. The last cell pellet was resuspended in calcium and magnesium-free Hank’s balanced salt solution with fetal bovine serum. Cells were further purified based on low forward and high side scatter using FACS to achieve a purity of > 95% based on morphological assessment.

Kidneys from zebrafish aged 3–6 months were dissected as previously described [74], pooled in HBSS (Sigma), homogenized, pelleted by centrifugation (250 g, 15 min), and gently resuspended in 6 mL HBSS. The suspension was gently layered on 2 mL of lymphocyte separation medium 1078 (Mediatech; CellGro, AK, USA) in a 15-mL Falcon tube and centrifuged (400 g, 30 min). The resulting layer of leukocytes was removed with a 1-mL sterile pipette and transferred to a 15-mL tube. HBSS was added to a total volume of 4 mL and leukocytes collected by centrifugation (400 g, 15 min). Yield assessed by hemocytometer cell counts was 1.1 ± 0.6 × 10^6^ cells/kidney (n = 15 independent preparations) and samples were 88.7 ± 6.2% (mean ± SD) granulocytes (n = 9 random fields).

Morphological confirmation of granulocytes was performed following Giemsa (human, mouse, dog, and zebrafish) or Wright staining (chicken) of cells smeared or spun onto poly-L-lysine slides.

### RNA isolation and mRNA-seq

Total RNA was isolated from granulocytes using Trizol (Invitrogen). The RNA quality was assessed using RNA 6000 Nano Chips on an Agilent Bioanalyzer (Agilent Technologies) to confirm an RNA integrity score of > 7.0. mRNA-seq was performed by Macrogen (Korea) using the Illumina Hi-Seq 2000 platform. RNA-seq libraries were prepared from > 1 μg of total RNA using TruSeq RNA sample prep kit (Illumina) according to the manufacturers’ instructions.

### Genome/gene sequences and gene structure annotations

Whole-genome assemblies of human, mouse, dog, chicken, and zebrafish (GRCh37.75, GRCm38.78, CanFam3.1.78, Galgal4.78, Zv9.78) were downloaded from Ensembl (Release 75). Intron sequences and gene structure information were retrieved from the UCSC Table browser [75]. Data on exon phases were retrieved via the ENSEMBL BioMart interface [76].

### RNA-seq data analysis and identification of IR events

Reference genome files were built and mRNA-seq reads were mapped to the respective reference genomes using STAR (Version 2.4) [77]. Details on sequencing depth and read mapping statistics for each sample are provided in Additional file 2: Table S5 and Figure S22. Gene body coverage was determined using the *geneBody coverage* module from the RSeQC package (v2.6.3) [78].

We used the IRFinder algorithm [26] for the detection of IR events in all known introns. IRFinder estimates the abundance of IR by computing the ratio between gene transcripts retaining an intron and the sum of all transcripts of the respective gene. We refer to this measure as the “IR ratio,” while others have also used the term “percent IR” (PIR) [2]. The IR ratio is in the range of 0–1; however, we only considered introns with an IR ratio ≥ 0.1. We excluded introns with insufficient splicing depth (<4 reads correctly crossing the splice junction) and insufficient coverage (splicing depth + trimmed mean intron depth < 10). IRFinder has a built-in routine to handle confounding factors. For example, partial IR resulting from splicing inside the intron is a distinct process and was not considered. On a per-gene basis, we considered the highest observed IR ratio for any of the retained introns as the gene’s IR ratio.

### Gene expression estimation

Gene expression levels specified as FPKM were determined using Cufflinks [79]. FPKM values for the genes in all species are specified in Additional file 4. Ratios of intron-retaining genes to all expressed genes were determined for all genes with an FPKM value ≥ 1.

### Annotation enrichment analysis

Gene annotation enrichment was performed using the Database for Annotation, Visualization and Integrated Discovery (DAVID 6.7) [80]. In order to make the analyses comparable across species, we used the set of orthologous genes shared among all species as background. Enriched terms with a *p* value < 0.05 were considered significant. Most commonly enriched terms are SwissProt and Protein Information Resource keywords (SP_PIR_KEYWORDS). All enriched functional annotations for each cluster of intron-retaining orthologs illustrated in Additional file 2: Figure S1 can be found in Additional file 3. The Circos plot in Fig. 1c was generated using the R package GOplot [81]. We have additionally performed a functional enrichment analysis of all intron-retaining genes using expressed genes as background in each species. The results are attached in Additional file 3.

### Gene orthologs

Orthologs of intron-retaining genes were determined based on orthology relationships extracted from the Ensembl BioMart interface. Venn diagrams were drawn with the VennDiagram R package.

### miRNA target site analysis

miRNA sequences and genomic coordinates were downloaded from the miRBase database (release 21) [82]. miRNA target site predictions were performed using miRanda v3.3a [83] with the energy threshold set to –14 kcal/mol and requiring strict alignment in the seed region (offset positions 2–8). Seed site enrichment analysis was performed using Sylamer [55], which calculates cumulative hypergeometric *p* values associated with small word occurrences in a sequence repository. 3′ UTR sequences of intron retaining genes were tested for 6mer seed site enrichment using the 3′ UTR sequences of all genes as background.

### Splice-site strength analysis

We used the maximum entropy model prosed in Yeo and Burge [37] to estimate the strengths of donor and acceptor sites in retained and non-retained introns.

### 3′ UTR introns

GTF files for all species under investigation were retrieved from the ENSEMBL ftp server (ftp.ensembl.org). Introns were flagged as 3′ UTR-based when located in the 3′ UTR of any of the transcripts of a gene.

### Statistical analyses

All statistical tests were performed using the statistical programming language R and are specified in the main text. In Fig. 2b, we used the geom_smooth function of the R ggplot2 package, which uses a generalized additive model with integrated smoothness estimation to fit a curve and standard error bounds to the intron length to IR ratio relationship.

IR profiles of orthologous genes in Additional file 2: Figure S1 were quantile normalized and grouped based on *k-means* clustering (with Euclidian distance). Species-based profiles (columns) were grouped using a hierarchical clustering approach (1-pearson average linkage). Clustering and heat map visualizations were performed using GENE-E (http://www.broadinstitute.org/cancer/software/GENE-E/).

## Additional files

Additional file 1: List of retained introns, their genomic coordinates, and IR ratios. (XLSX 1965 kb)

Additional file 2: Supplementary tables and figures. (DOCX 6927 kb)

Additional file 3: Sheets 1–7: All enriched functional annotations for each cluster of intron-retaining orthologs illustrated in Additional file 2: Figure S1. Sheet 8: List of homologous genes used as background. Sheets 9–13: All enriched functional annotations of all intron-retaining genes using expressed genes as background in each species. (XLSX 913 kb)

Additional file 4: Gene expression levels (FPKM) in all species. (XLSX 16883 kb)

Additional file 5: Scalable version of Fig. 1c. (PDF 8057 kb)
