# Supplementary material for: Intron retention enhances gene regulatory complexity in vertebrates
Source: Genome Biol. 2017 Nov 16;18:216. doi: 10.1186/s13059-017-1339-3 (PMC5688624; doi:10.1186/s13059-017-1339-3)
Supplement: Supplementary file 2 — Supplementary tables and figures. (DOCX 6927 kb) [file 13059_2017_1339_MOESM2_ESM.docx]

# Supplemental Material

**Intron retention enhances gene regulatory complexity in vertebrates**

Ulf Schmitz^1,2^, Natalia Pinello^1,2,3^, Fangzhi Jia^1,2^, Sultan Alasmari^4^, William Ritchie^5^, Maria-Cristina Keightley^4^, Shaniko Shini^6^ , Graham J Lieschke^4^, Justin J-L Wong^1,2,3,‡^, John EJ Rasko^1,2,7, ‡,*^

^1^Gene & Stem Cell Therapy Program, Centenary Institute, University of Sydney, Camperdown 2050, Australia, ^2^Sydney Medical School, University of Sydney, Camperdown 2050, Australia, ^3^Gene Regulation in Cancer Laboratory, Centenary Institute, University of Sydney, Camperdown 2050, Australia, ^4^Australian Regenerative Medicine Institute, Monash University, Clayton, Victoria 3800, Australia, ^5^CNRS, UMR 5203, Montpellier 34094, France, ^6^School of Biomedical Sciences, The University of Queensland, Brisbane QLD 4072, Australia, ^7^Cell and Molecular Therapies, Royal Prince Alfred Hospital, Camperdown 2050, Australia

**^; ‡^** These authors contributed equally

**^*^** Correspondence and requests for materials should be addressed to:

John EJ Rasko, Locked Bag 6, Newtown, NSW 2042 Australia; Phone: +61 2 9565 6156; Fax: +61 2 9565 6101; Email: j.rasko@centenary.org.au

## Supplementary Tables

| **Study** | **Purified cell type or comparison** | **Enriched functional annotations** | |
| --- | --- | --- | --- |
| Edwards *et al.* 2016 [1]  (mouse+human) | megakaryocyte-erythrocyte precursors vs. erythrocytes | **IR**^🡹^ | **IR_🡻_** |
|  |  | mitosis, chromatin, and the spindle | ribosomes, translation, splicing, and adenosine triphosphate biosynthesis |
|  | megakaryocyte-erythrocyte precursors vs. megakaryocytes | **all diff IR genes, most have IR_🡻_** | |
|  |  | actin, immunity, ncRNA, endocytosis, and the proteasome | |
| Ni *et al.* 2016 [2]  (human) | CD4+ T cell activation (upregulated genes with reduced IR and Pol II unchanged) | **upregulated genes with IR_🡻_ and Pol II unchanged** | |
|  |  | proteasome complex, NF-KB, Negative regulation of transcription, Apoptosis, Caspase3 activation signaling, West nile virus, Regulation of eif2, validated targets of C-MYC | |
| Pimentel *et al.* 2016 [3]  (human) | terminal erythropoeisis (proerythroblasts,  early/late basophilic erythroblasts,  polychromatophilic erythroblasts  and orthochromatophilic erythroblasts) | **Clusters of IR profiles during differentiation** | |
|  |  | **IR**^🡹^ **(Clusters 1 and 2)** | **IR**^🡺^ **(Clusters 4 and 5)** |
|  |  | RNA splicing, mRNA, metabolic process, transcription, RNA binding, membrane organization, nuclear export, chromosome organization,cell death, regulation of I-kappaB kinase/NF-kappaB cascade, cell cycle | zinc ion binding, enzyme binding, regulation of transcription,  cation binding, RNA splicing |

Table S1 Enriched functional annotations in purified hematopoietic cells other than granulocytes.

|  | **Median** | | **Mean** | |
| --- | --- | --- | --- | --- |
| **Species** | **All** | **IR** | **All** | **IR** |
| Human | 1,677 | 340 | 6,566 | 792 |
| Mouse | 1,402 | 374 | 5,355 | 848 |
| Dog | 1,091 | 351 | 3,954 | 752 |
| Chicken | 797 | 560 | 2,819 | 930 |
| Zebrafish | 1,023 | 366 | 3,092 | 1,083 |

Table S2 Median and mean lengths of retained and all other introns. All values were rounded to the nearest whole number.

|  | **Intron-retaining genes (% of expr. genes)** | **Number of retained introns** | **Mean length (nt)** | **GC (%)** | | | **Density (retained introns per kbp exon sequence)** |  |
| --- | --- | --- | --- | --- | --- | --- | --- | --- |
| **Human** | 17.4 | 5,463 | 792.4 | | 54.1 | 0.15 | | |
| **Mouse** | 13.6 | 5,157 | 848.1 | | 50.8 | 0.13 | | |
| **Dog** | 18.6 | 6,337 | 752.0 | | 54.6 | 0.18 | | |
| **Chicken** | 40.8 | 13,892 | 930.3 | | 46.8 | 0.53 | | |
| **Zebrafish** | 7.8 | 2,325 | 1,083.1 | | 37.3 | 0.05 | | |

Table S3 Description of intron-retaining genes and retained introns. pc = protein-coding

| **Species** | **Seed site** | **Predicted regulatory miRNAs** |
| --- | --- | --- |
| Human | TATTTA | dre-miR-10b-2-3p, hsa-miR-3121-3p, hsa-miR-5692a, mmu-miR-3473f |
|  | TTTTTA | hsa-miR-548c-3p, hsa-miR-548d-3p, dre-miR-740, hsa-miR-548j-3p, hsa-miR-548h-3p, hsa-miR-548x-3p, hsa-miR-548z, hsa-miR-548ac, hsa-miR-548ae-3p, hsa-miR-548ah-3p, hsa-miR-548aj-3p, hsa-miR-548am-3p, hsa-miR-548aq-3p, mmu-miR-7119-3p, hsa-miR-548bb-3p |
|  | AATAAA | mmu-miR-325-3p, gga-miR-6701-3p, mmu-miR-126b-5p |
|  | AAAAAA | mmu-miR-129-5p, hsa-miR-129-5p, dre-miR-129-5p, dre-miR-722, dre-miR-737-5p, dre-miR-737-5p, cfa-miR-129, mmu-miR-6951-3p, mmu-miR-6951-3p, mmu-miR-7116-3p, mmu-miR-7116-3p |
|  | TTTGTT | hsa-miR-7-1-3p, hsa-miR-7-2-3p, mmu-miR-7a-1-3p, hsa-miR-495-3p, mmu-miR-495-3p, mmu-miR-1192, cfa-miR-495, hsa-miR-5688 |
|  | TATATA | hsa-miR-190a-3p, hsa-miR-410-3p, gga-miR-466, mmu-miR-344d-3p, mmu-miR-669b-3p, mmu-miR-669f-3p, gga-miR-1812-5p, cfa-miR-410, mmu-miR-344e-3p, gga-miR-2188-3p, hsa-miR-5011-5p |
| Mouse | TTTTAT | hsa-miR-3163 |
|  | TTTATA | mmu-miR-142a-5p, hsa-miR-142-5p, mmu-miR-340-5p, hsa-miR-340-5p, dre-miR-142a-5p, dre-miR-142b-5p, mmu-miR-466l-3p, cfa-miR-340, hsa-miR-3163, hsa-miR-5590-3p |
|  | AATAAA | mmu-miR-325-3p, gga-miR-6701-3p, mmu-miR-126b-5p |
|  | TTTTTA | hsa-miR-548c-3p, hsa-miR-548d-3p, dre-miR-740, hsa-miR-548j-3p, hsa-miR-548h-3p, hsa-miR-548x-3p, hsa-miR-548z, hsa-miR-548ac, hsa-miR-548ae-3p, hsa-miR-548ah-3p, hsa-miR-548aj-3p, hsa-miR-548am-3p, hsa-miR-548aq-3p, mmu-miR-7119-3p, hsa-miR-548bb-3p |
|  | TATTTA | dre-miR-10b-2-3p, hsa-miR-3121-3p, hsa-miR-5692a, mmu-miR-3473f |
|  | TTTGTT | hsa-miR-7-1-3p, hsa-miR-7-2-3p, mmu-miR-7a-1-3p, hsa-miR-495-3p, mmu-miR-495-3p, mmu-miR-1192, cfa-miR-495, hsa-miR-5688 |
| Dog | ATAAAT | gga-miR-1460 |
|  | TATTTA | dre-miR-10b-2-3p, hsa-miR-3121-3p, hsa-miR-5692a, mmu-miR-3473f |
|  | AATAAA | mmu-miR-325-3p, gga-miR-6701-3p, mmu-miR-126b-5p |
|  | ATTTAT | mmu-miR-466l-3p |
|  | TTTGTA | hsa-miR-524-5p, hsa-miR-520d-5p, mmu-miR-8118 |
|  | TAAAAA | hsa-miR-2681-5p |
| Chicken | AACAGT | hsa-miR-452-5p, cfa-miR-452, hsa-miR-4676-3p, hsa-miR-892c-3p, gga-miR-7448-3p |
|  | ACACTA | hsa-miR-648, hsa-miR-4255, hsa-miR-4280, mmu-miR-6358, mmu-miR-6360, mmu-miR-6376, cfa-miR-8861 |
|  | CGTCGC | gga-miR-1634 |
|  | TTTGTA | hsa-miR-524-5p, hsa-miR-520d-5p, mmu-miR-8118 |
|  | CAATTG | gga-miR-1416-3p |
| Zebrafish | AGTGAG | hsa-miR-1304-3p, mmu-miR-6939-3p, mmu-miR-6992-3p, mmu-miR-7015-3p |
|  | TAGCCT | mmu-miR-3099-3p |
|  | GTTAAA | hsa-miR-302c-5p, dre-miR-27c-5p, hsa-miR-552-5p, gga-miR-302c-5p, cfa-miR-302c |
|  | CTAGCC | hsa-miR-6069 |
|  | ACTAGC | hsa-miR-8086 |

Table S4 Top enriched seed sites in 3′ UTR sequences of intron-retaining genes and predicted regulatory miRNAs.

|  | **Human** | **Mouse** | **Dog_1** | **Dog_2** | **Chick_1** | **Chick_2** | **Zebraf_1** | **Zebraf_2** |
| --- | --- | --- | --- | --- | --- | --- | --- | --- |
| **Number of input reads (x 10^6^)** | 65.0 | 65.8 | 76.1 | 72.0 | 32.6 | 66.0 | 63.9 | 70.2 |
| **Uniquely mapped reads number (x 10^6^)** | 61.4 | 62.6 | 71.3 | 67.4 | 28.5 | 57.3 | 59.4 | 64.7 |
| **Uniquely mapped reads (%)** | 94.38 | 95.13 | 93.73 | 93.66 | 87.20 | 86.84 | 92.95 | 92.14 |
| **Total number of splices (x 10^6^)** | 35.6 | 40.5 | 38.8 | 37.6 | 14.5 | 30.1 | 45.4 | 48.0 |
| **Mismatch rate per base (%)** | 0.45 | 0.32 | 0.39 | 0.40 | 0.59 | 0.56 | 0.38 | 0.39 |

Table S5 Mapping statistics.

## Supplementary Figures


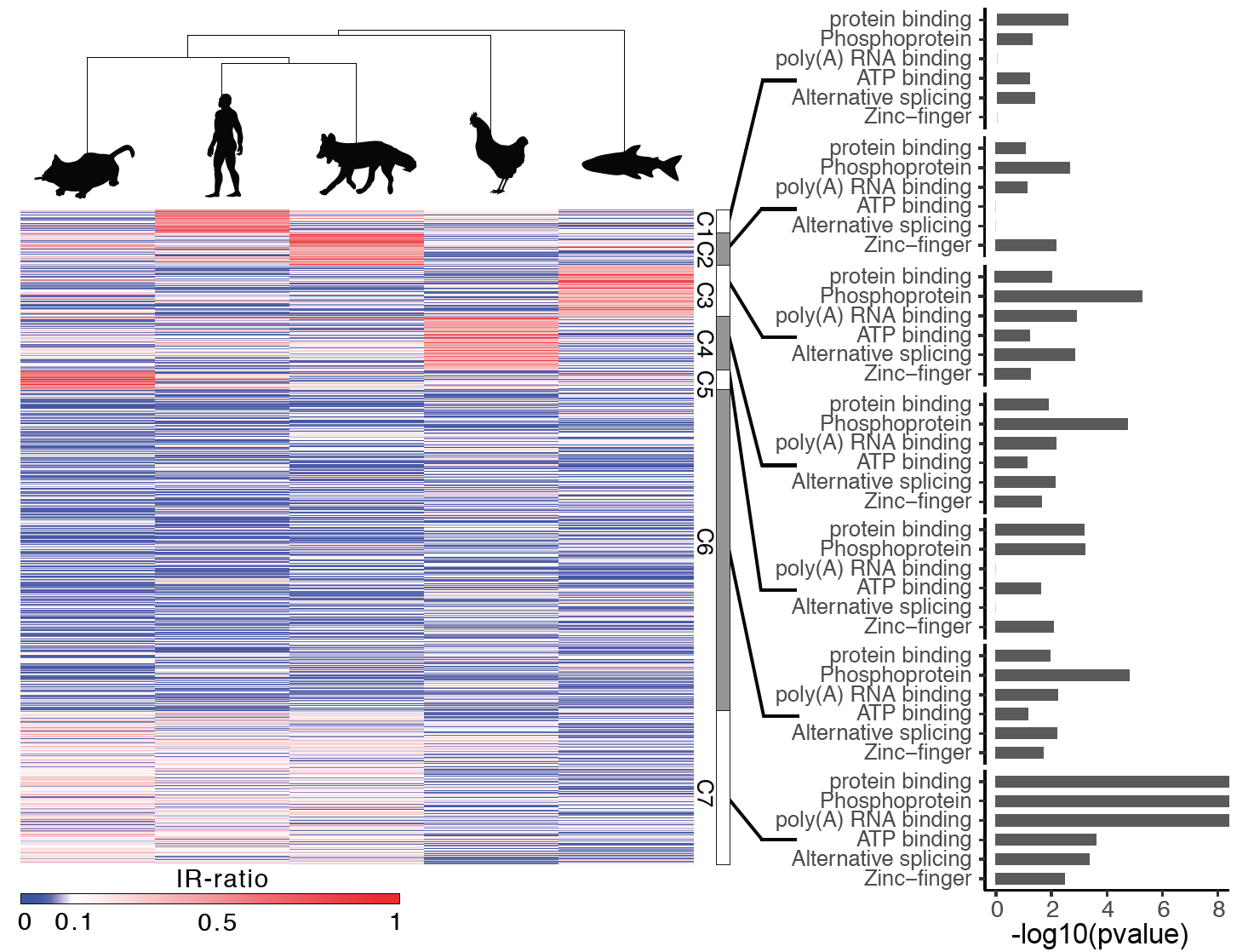


Figure S1 Heat map showing clusters (C1-C7) of species-specific IR ratio profiles and annotations reoccurring in association with the genes in each cluster. Each gene in the heat map has a retained intron in at least one species, i.e. non intron-retaining genes were excluded.


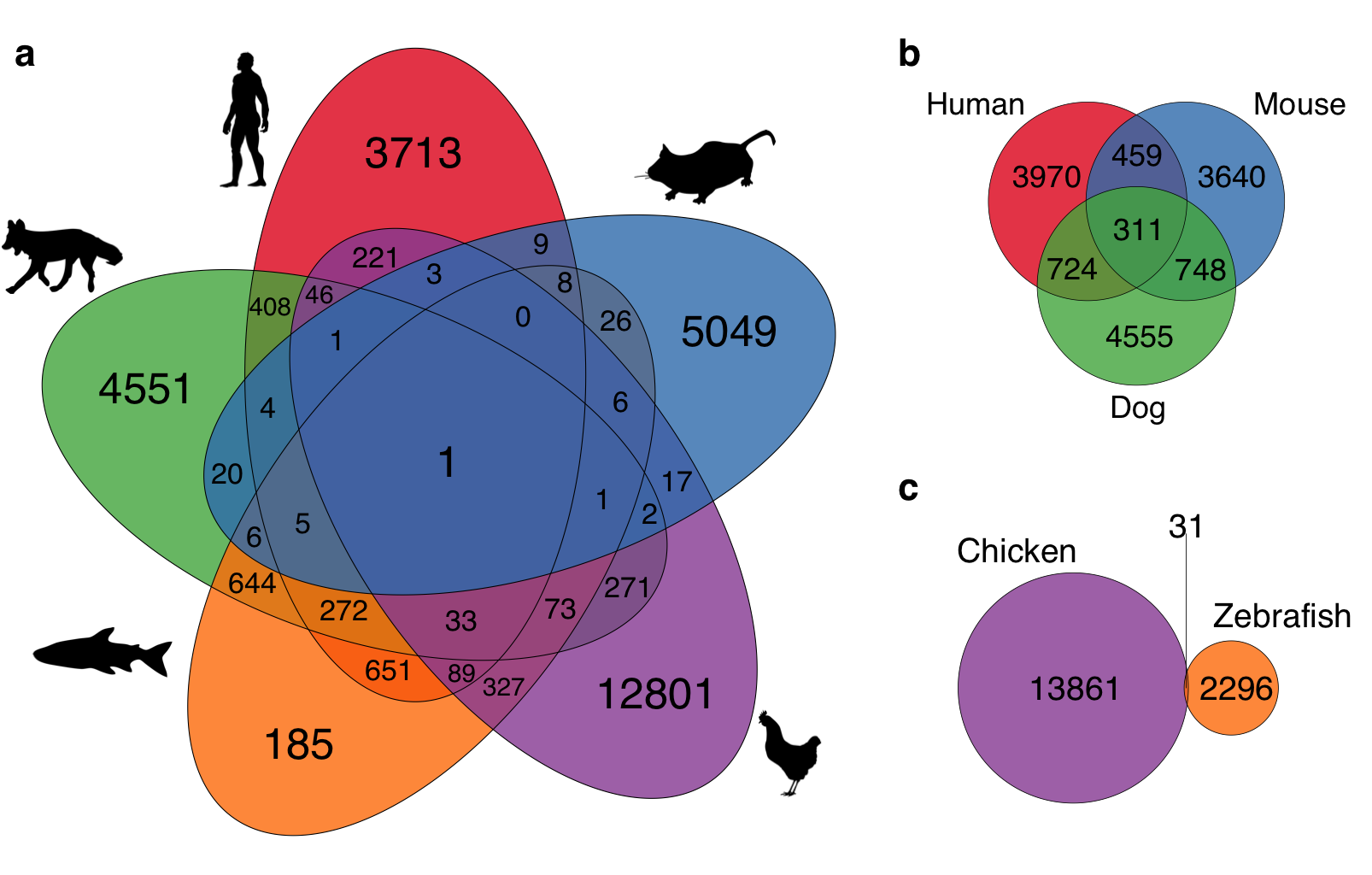


Figure S2 IR conservation in mammalian and vertebrate species. (a) The 5-way Venn diagram shows the intersections of orthologous retained introns between species. (b) The 3-way Venn diagram shows the intersecting IR orthologs in placental mammals (human, mouse, dog), and (c) the 2-way Venn diagram illustrates the intersection of IR orthologs in the non-placental vertebrates (chicken and zebrafish). Orthologous introns were obtained by converting the genomic coordinates between genomes using the liftOver tool from Galaxy [4].

Figure S3 Expression analyses of genes with and without IR. Box plots show a comparison of FPKM values of expressed intron-retaining genes (IR) vs. expressed non intron-retaining genes (Other). IR affects a whole range of transcripts, from highly abundant genes (for fine-tuning) to lowly expressed genes (for minimal expression or silencing). The spectrum of IR transcript abundance is therefore similar to that of non-IR affected transcripts and IR-transcripts that evade NMD.

Figure S4 Length of retained introns. Box plots showing the log10 lengths of non-retained (left) and retained introns (right). The dashed purple horizontal lines represent the median lengths of chicken introns (upper) and retained introns, which are shorter and longer than in all other species respectively. Plots are truncated and outliers are not shown.

Figure S5 Probability density function of the IR ratio in retained introns.

Figure S6 Phases of retained introns. (a) Schematic representation of intron phases. A phase 0 intron is embedded between two exons that end and start with a complete codon. A phase 1 intron intercepts a codon, where the 1^st^ nucleotide remains with the upstream exon, and the 2^nd^ and 3^rd^ nucleotides of the codon are provided in the downstream exon. In case of a phase 2 intron two nucleotides remain with the upstream exon and the 3^rd^ nucleotide of that codon marks the beginning of the downstream exon. (b) Bar plots illustrating the intron phase distribution for all introns (light color, excluding retained introns) and retained introns (dark color). The intron phase distribution is slightly but significantly shifted away from phase 0 in retained introns in all five species (Chi-squared goodness of fit test, p< 2.2e-09).


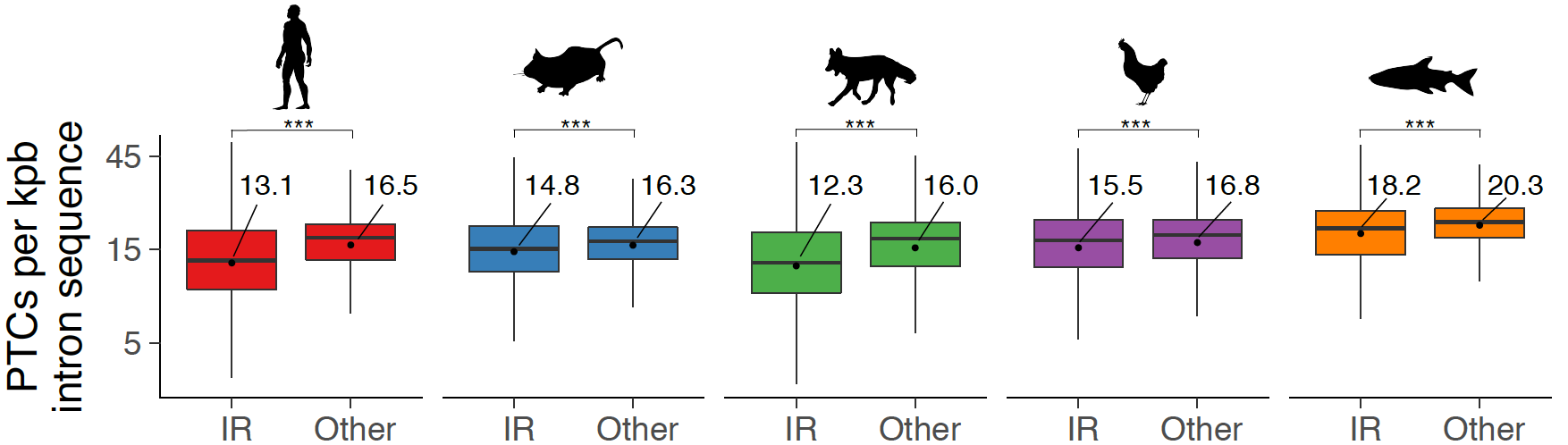


Figure S7 Boxplots showing the number of PTCs per kbp intron sequence. The number of PTCs per kbp intron sequence in retained introns (IR) is slightly but significantly lower than in all other introns (Other). Mann Whitney U test; *** (p < 2.2e-16). PTC = premature termination codon; outliers are not shown.


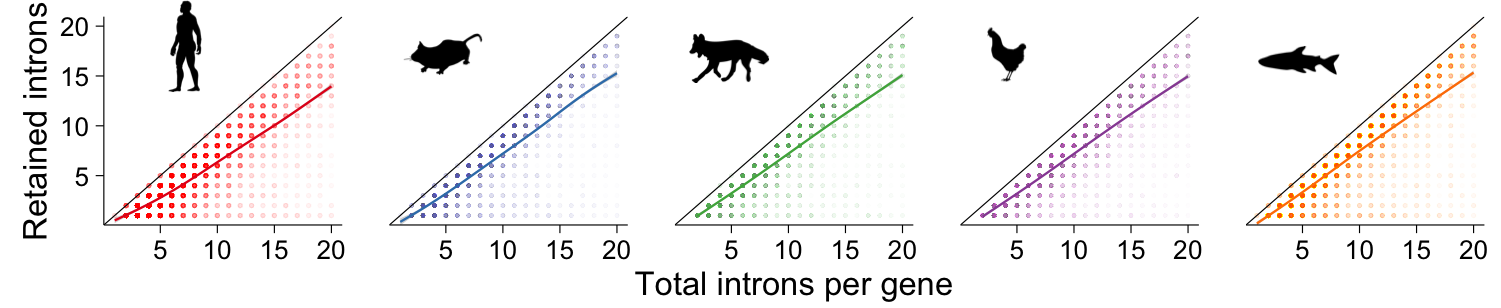


Figure S8 Dot plots illustrating the linear relationship between the number of introns in a gene and the number of retained introns. Dots with higher opacity reflect a higher frequency of transcripts with the same ratio of retained introns to the total number of introns in a transcript.

Figure S9 Distribution of the relative position of retained introns in the transcript. (a) Probability density functions of the position of retained introns in relation to other introns in the gene structure (see also Figure 5a – main manuscript). The top row presents probability densities for very long introns (with a length that is 3 standard deviations above the mean intron length) in each species. Below are the densities for all introns shorter than that. The third row presents introns with very high IR-ratios (>0.8) and below all other introns are represented. (b) Gene body coverage analysis of mapped reads.


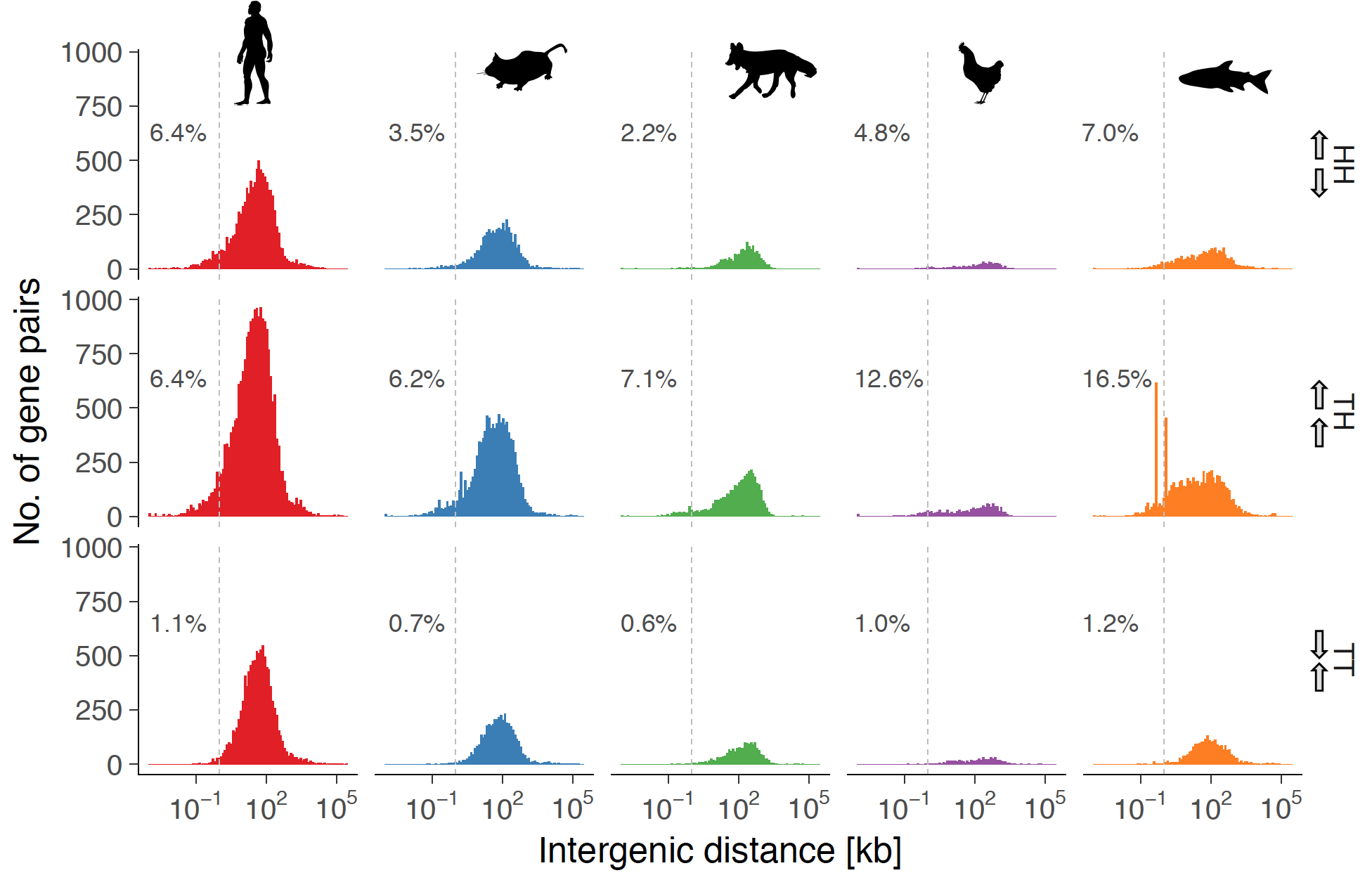


Figure S10 Histograms of binned intergenic distances between genes that do not retain introns.

Figure S11 Probability of IR events based on the number of introns. The figure shows the estimated posterior probability (*y-axis*) of observing an IR event in a transcript with a certain number of introns (*x-axis*) determined using the Bayes' rule.


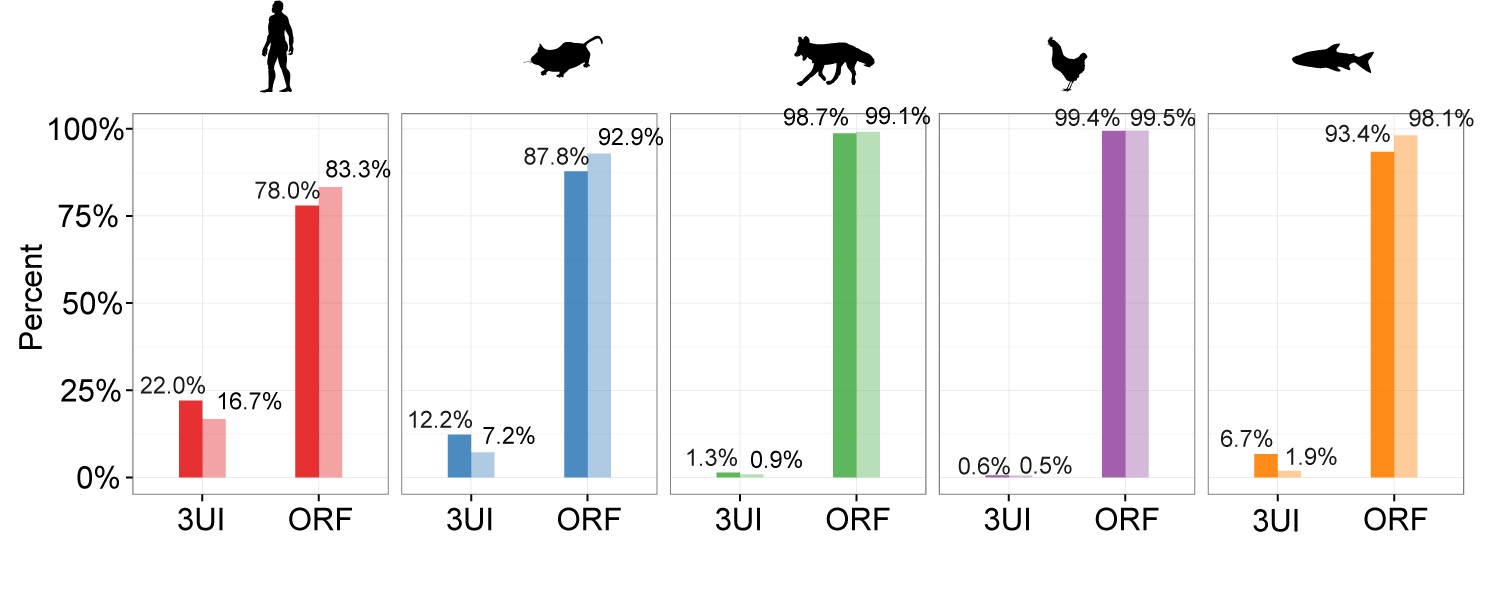


Figure S12 Retention of 3′ UTR introns. The bar chart shows the fraction of introns located in the 3′ UTR (3UI) or open reading frame (ORF) of a gene in the groups of retained (dark color) and non-retained introns (light color), respectively.

Figure S13 MiRNA recognition element (MRE) density. Comparison of the number of predicted miRNA binding sites per kilobase intron sequence in retained and other introns. The fine diagonal indicates equivalence between predicted miRNA binding sites in retained and non-retained introns. Non-retained introns (other introns) include lowly and non-expressed introns.


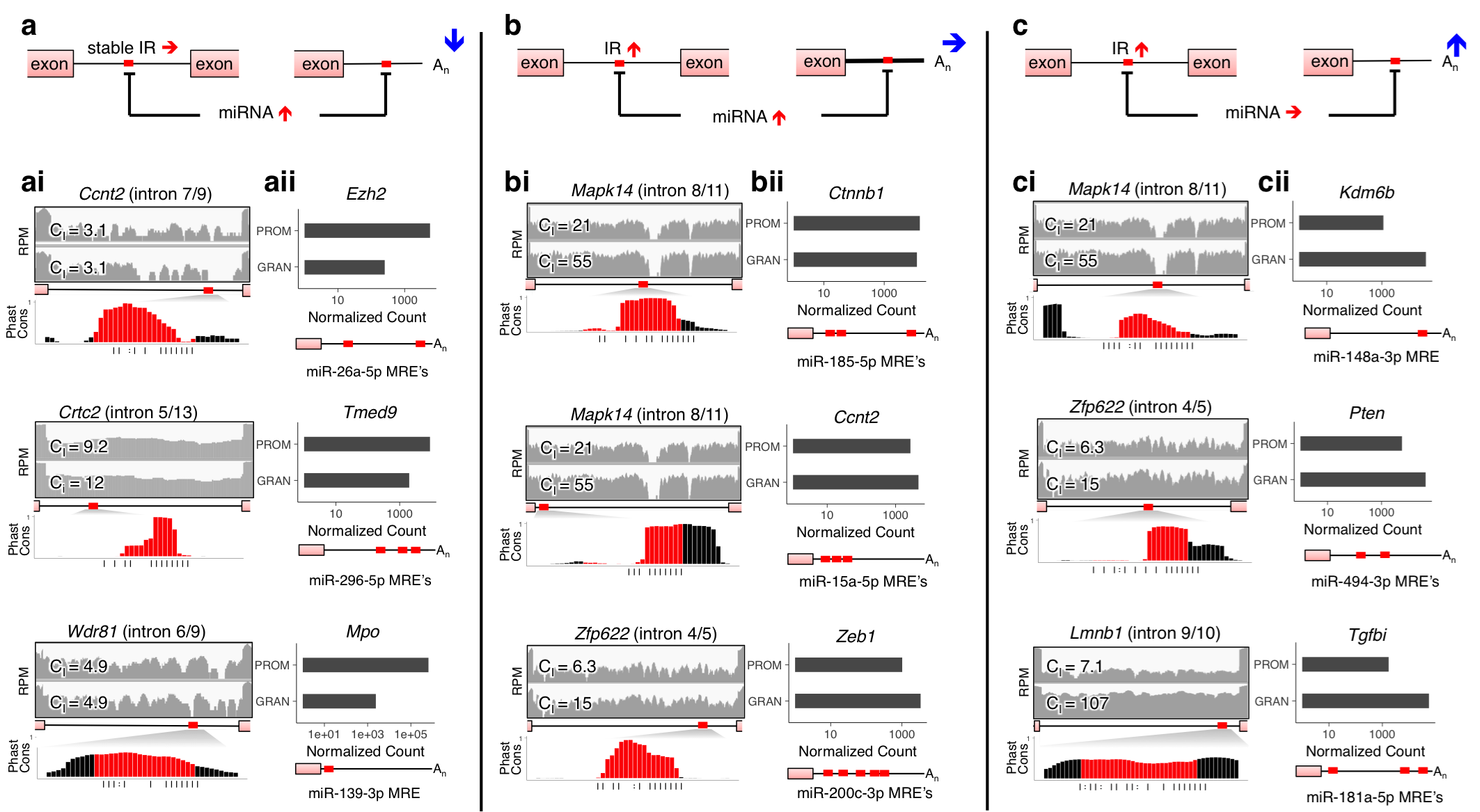


Figure S14 Illustrative examples of putative miRNA sponge introns. Each column in this figure shows a sketch and three illustrative examples of different forms of the regulatory interplay (crosstalk) between retained introns and two target genes both harboring binding site(s) for the same miRNA. The blue arrows in each sketch indicate the expected expression change of the endogenous miRNA targets. (a) The three genes shown in ai contain introns that are stably retained in granulocytes and promyelocytes and harbor one ore more predicted binding sites for a miRNA with increased expression (fold change > 2) in terminally differentiated granulocytes. Thus the left panel acts as a ‘control’ arm. The bar plots in aii show that another target of the same miRNA, with one or more binding sites in the 3′ UTR, are downregulated. This downregulation might be realized by excess miRNAs that are not sponged by the retained introns (because the level of the other does not change). (b) The three genes shown in bi contain introns with increased IR in granulocytes and harbor one or more binding sites of upregulated miRNAs. The bar plots in bii show that the expression of another target of the same miRNAs, with one or more binding sites in the 3′ UTR, remains unchanged. The rise in IR might enhance the sponge effect, which counterweighs the increased number of available miRNAs. (c) The three genes shown in ci contain introns with increased IR in granulocytes and harbor one or more binding sites of uniformly expressed miRNAs. The bar plots in cii show that the expression of another target of the same miRNAs, with one or more binding sites in the 3′ UTR, is upregulated. The rise in IR might increase competition for available miRNAs whereby other targets are relieved from miRNA-induced repression. mRNA-seq reads (normalized per total number of mappable reads, RPM) in promyelocytes (PROM), and granulocytes (GRAN) are portrayed on the same scale using IGV v2.1 software. The bar plots beneath the sequencing coverage plots indicate the conservation of the putative miRNA binding sites (red) ±10 nt (black) in the introns (PhastCons score). The 3′ UTR sketches beneath the count data of the endogenous miRNA targets indicate the number and loci of predicted binding sites of the crosstalking miRNA. All endogenous miRNA targets, except for *Mpo*, have been experimentally validated based on data from the miRTarBase database [5]. Data were obtained from our previous study comparing promyelocyte against granulocyte transcriptomes in mice (GEO accession numbers: GSE48307 [6], GSE57624 [7]).


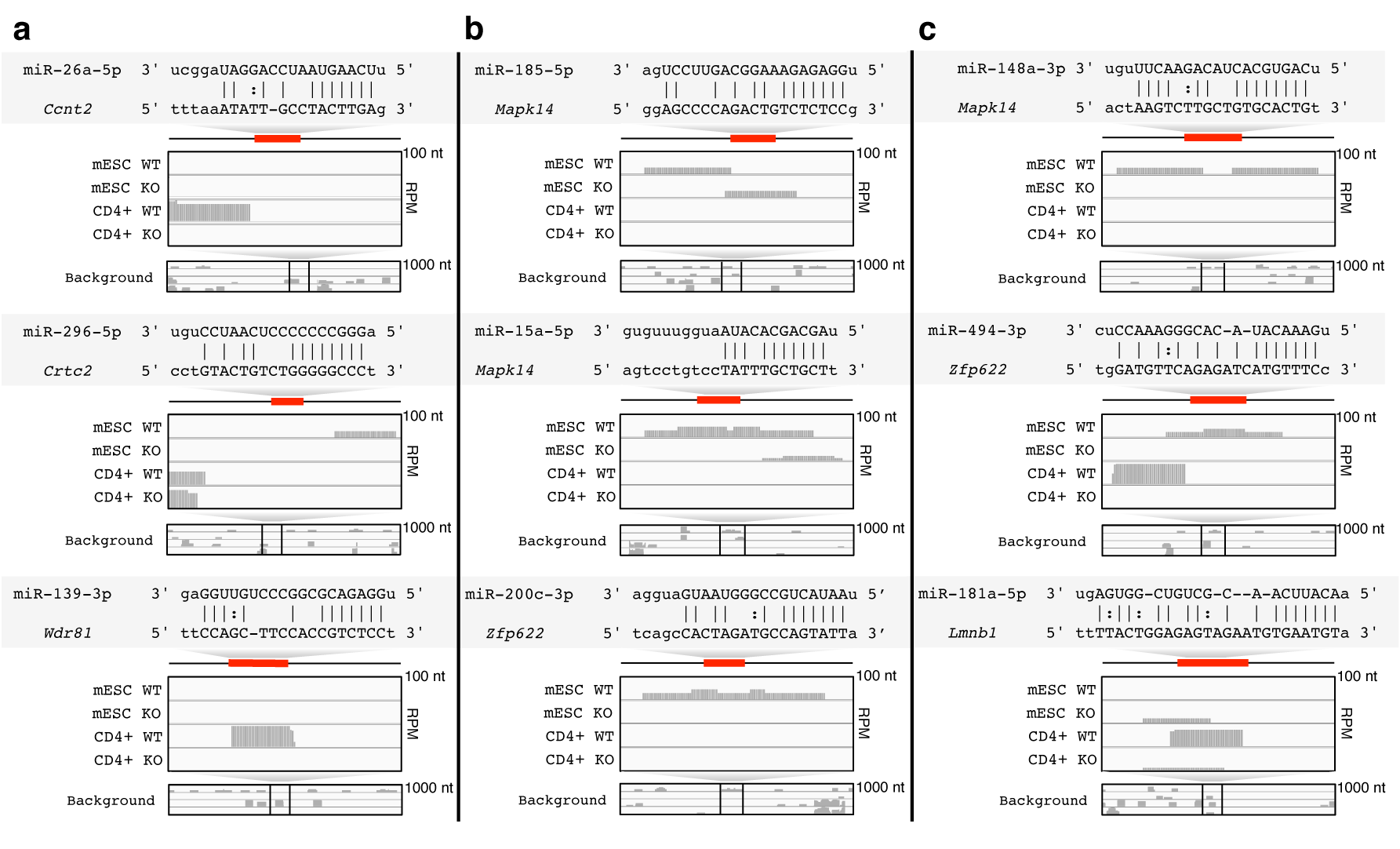


Figure S15 Argonaute HITS-CLIP reads map to predicted MREs in retained introns. For each example in Figure S14 this figure shows predicted binding patterns of miRNAs to MREs in introns (grey boxes). Below each alignment, coverage plots illustrate mapped reads of Argonaute HITS-CLIP data in the vicinity of the predicted MREs. A coverage plot of the larger background (1000 nt) is also provided. HITS-CLIP experiments have been performed in wild type mouse embryonic stem cells (mESC WT), Dicer depleted mESCs (mESC KO), wild type mouse CD4+ T cells (CD4+ WT), and miRNA-155 depleted CD4+ T cells (CD4+ KO). The data was retrieved from Gene Expression Omnibus (GEO accession numbers: GSE25310 [8], GSE41288 [9]).


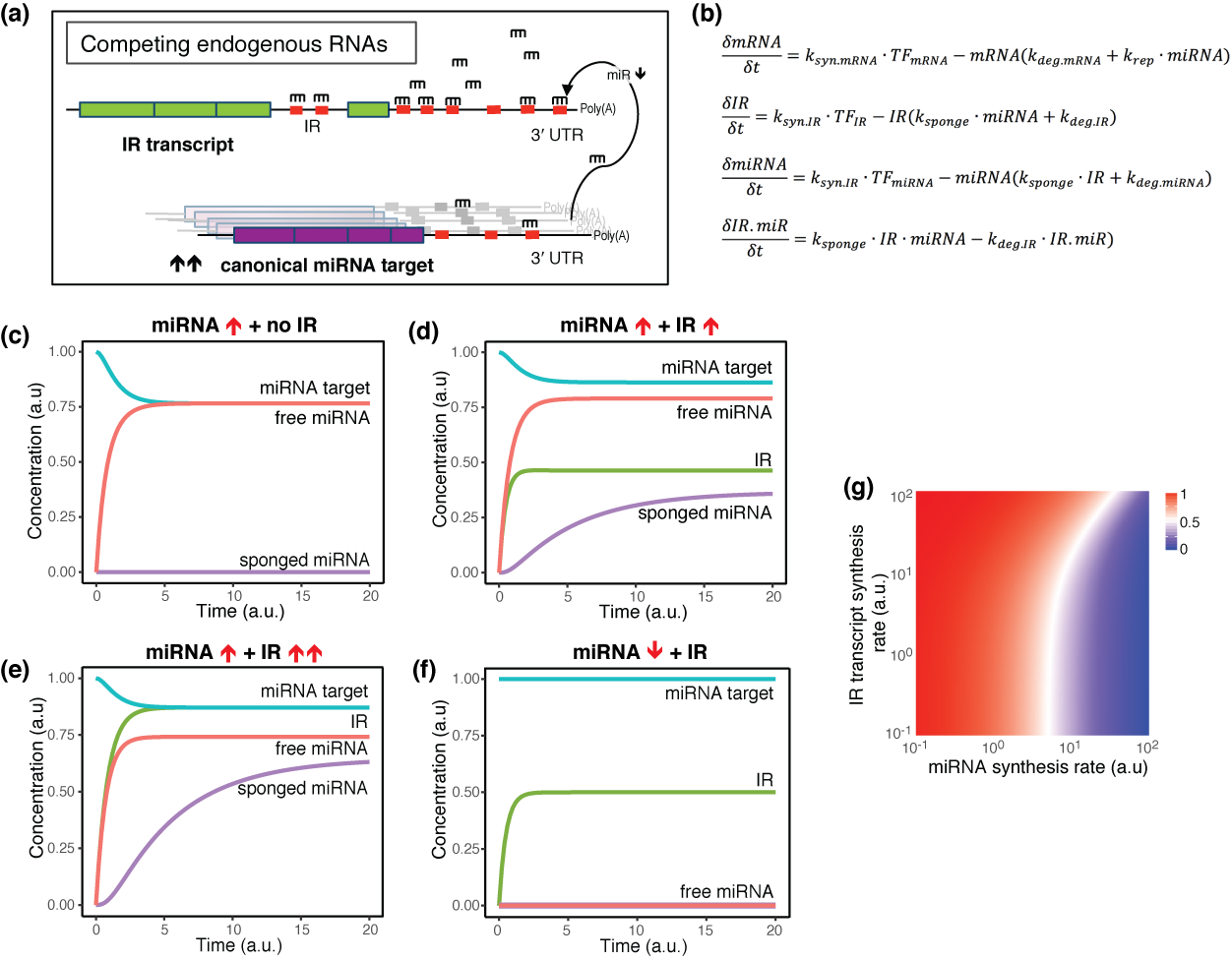


Figure S16 Phenomenological model of intron-retaining transcripts acting as competing endogenous RNA. (a) Illustration of the putative miRNA sponge mechanism realized by intron-retaining transcripts. (b) A kinetic model composed of ordinary differential equations that reflect the time-dependent dynamics of molecules/complexes involved in the IR-induced miRNA sponge mechanism. The model accounts for concentration changes of the miRNA target (1st equation), intron-retaining transcripts (2nd equation), the miRNA (3rd equation), and the miRNA/IR complex (4th equation). (c-f) Model simulations showing dynamics of miRNA target concentration based on different IR and miRNA expression scenarios (bottom right). (g) Contour plot showing steady state miRNA target concentrations (arbitrary units normalized to values between 0 and 1) for varying IR transcript and miRNA synthesis rates in intervals [10^-1^…10^2^]. a.u. = arbitrary units.

Figure S17 Sylamer [10] enrichment plots illustrating seed motifs enriched in the 3′ UTR sequences of intron-retaining genes. The top 5 enriched motifs for each species are colored in the enrichment plots. The canonical polyadenylation signal (AATAAA), which is enriched in human, mouse, and dog, is not highlighted. The table on the bottom left lists motifs that are among the top 5 motifs in more than one species.


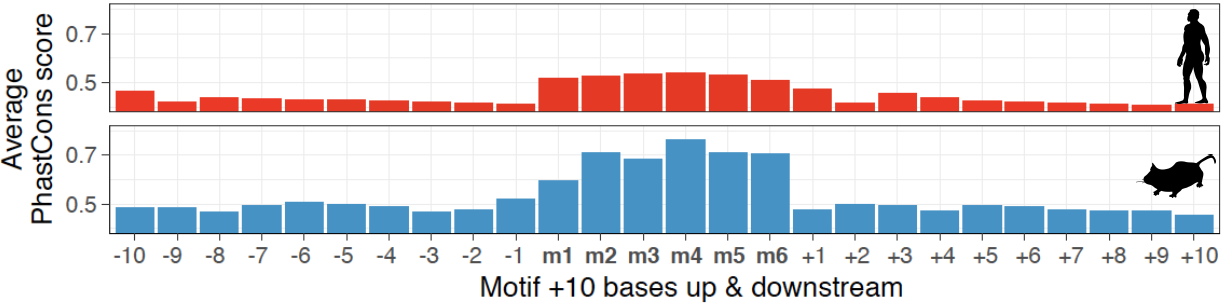


Figure S18 Conservation of motifs over-represented in 3′ UTR sequences of intron-retaining genes. Depicted are the average PhastCons scores of 1,000 randomly selected occurrences of mutually over-represented motifs in human and mouse as well as their flanking regions (±10 nt). Mutually over-represented motifs are: TATTA, TTTGTT, and TTTTTA. Nucleotides of enriched motifs (m1-m6) have significantly higher scores than those in the flanking regions (Wilcoxon rank sum test p-value < 2.2e-16). The scores were retrieved from the UCSC PhastCons100way and PhastCons60way tables.

Figure S19 IR-mediated fine-tuning of gene expression and network dynamics. The illustrations depict possible consequences of IR-mediated gene regulation that add to the gene regulatory complexity of a cell. (a) Orthotopic IR may serve to regulate overexpressing genes towards levels that are desired for a particular phenotype. (b) In this scenario orthotopic IR causes target repression towards ineffective levels (the target is switched off). (c) The effect of IR in a cascade of sequential repression. (d) In a gene regulating a double positive feedback loop IR may induce a memory effect causing the loop to lock irreversibly into a steady state (expression of B and C is activated). a.u. = arbitrary unit.

Figure S20 Sequence motifs overrepresented in the 3′ UTRs of genes upregulated in hematopoietic differentiation. Significance landscape plot showing that most of the enriched sequence motifs in 3′ UTRs of intron-retaining genes are also overrepresented in genes that are upregulated during hematopoietic differentiation. The canonical polyadenylation signal (AATAAA), which is also enriched, is not highlighted. Upregulated genes were derived from mouse proteomics data comparing protein expression in granulocytes versus promyelocytes in our previous study [11, 12].


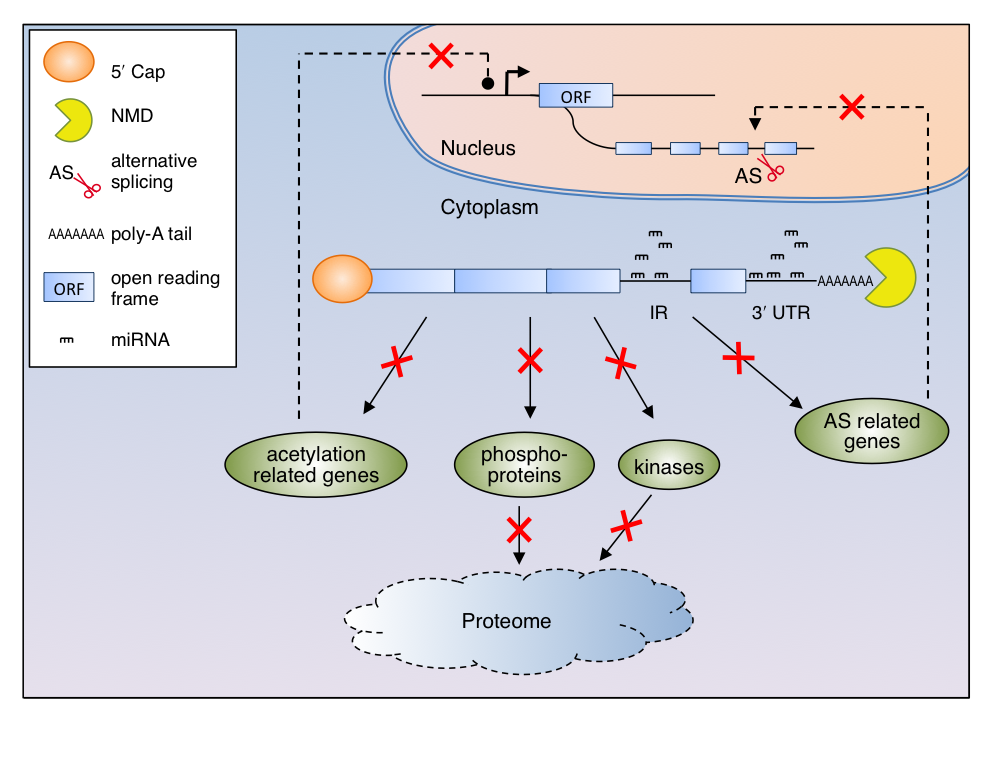


Figure S21 Consequences of IR in differentiated granulocytes. Theilgaard-Mönch *et al.* observed a general decline of proliferative and general cellular activity during terminal granulocytic differentiation [13]. According to our results, IR-mediated gene regulation may be involved in processes leading to this observation.


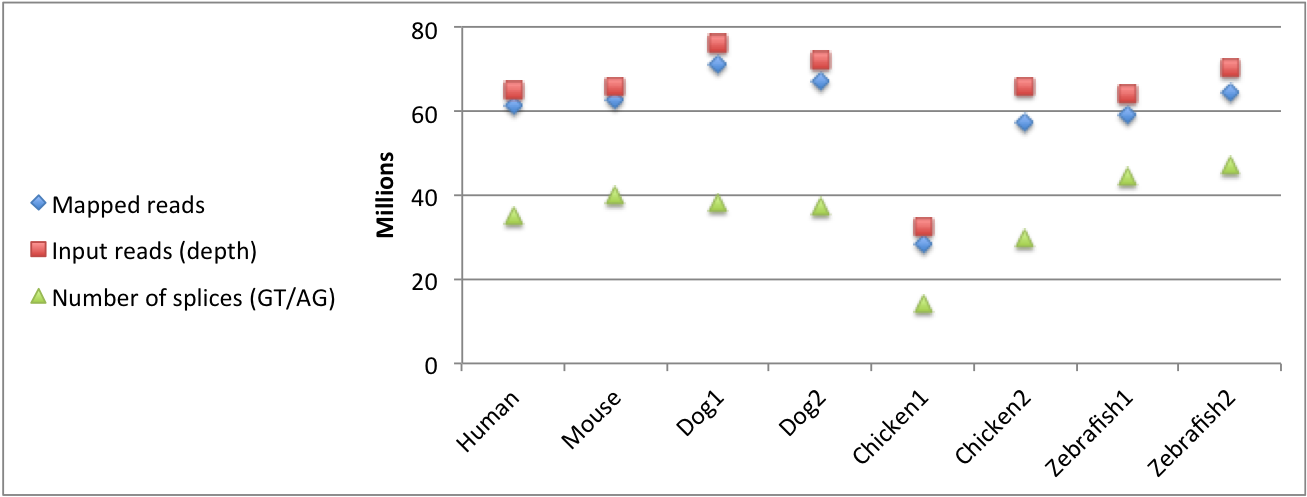


Figure S22 Read mapping statistics.

1. Edwards CR, Ritchie W, Wong JJ, Schmitz U, Middleton R, An X, Mohandas N, Rasko JE, Blobel GA: **A dynamic intron retention program in the mammalian megakaryocyte and erythrocyte lineages.** *Blood* 2016.

2. Ni T, Yang W, Han M, Zhang Y, Shen T, Nie H, Zhou Z, Dai Y, Yang Y, Liu P, et al: **Global intron retention mediated gene regulation during CD4+ T cell activation.** *Nucleic Acids Res* 2016.

3. Pimentel H, Parra M, Gee SL, Mohandas N, Pachter L, Conboy JG: **A dynamic intron retention program enriched in RNA processing genes regulates gene expression during terminal erythropoiesis.** *Nucleic Acids Res* 2016, **44:**838-851.

4. Hinrichs AS, Karolchik D, Baertsch R, Barber GP, Bejerano G, Clawson H, Diekhans M, Furey TS, Harte RA, Hsu F, et al: **The UCSC Genome Browser Database: update 2006.** *Nucleic Acids Res* 2006, **34:**D590-598.

5. Chou CH, Chang NW, Shrestha S, Hsu SD, Lin YL, Lee WH, Yang CD, Hong HC, Wei TY, Tu SJ, et al: **miRTarBase 2016: updates to the experimentally validated miRNA-target interactions database.** *Nucleic Acids Res* 2016, **44:**D239-247.

6. Wong JJ, Ritchie W, Ebner OA, Selbach M, Wong JW, Huang Y, Gao D, Pinello N, Gonzalez M, Baidya K, et al: **Orchestrated intron retention regulates normal granulocyte differentiation.** Gene Expression Omnibus: https://www.ncbi.nlm.nih.gov/geo/query/acc.cgi?acc=GSE48307; 2013.

7. Wong JJ, Ritchie W, Gao D, Lau KA, Gonzalez M, Choudhary A, Taft RJ, Rasko JE, Holst J: **Identification of nuclear-enriched miRNAs during mouse granulopoiesis.** Gene Expression Omnibus: https://www.ncbi.nlm.nih.gov/geo/query/acc.cgi?acc=GSE57624; 2014.

8. Leung AK YA, Bhutkar A, Zheng GX, Bosson AD, Nielsen CB, Sharp PA: **Genome-wide identification of Ago2 binding sites from mouse embryonic stem cells with and without mature microRNAs.** Gene Expression Omnibus: https://www.ncbi.nlm.nih.gov/geo/query/acc.cgi?acc=GSE25310; 2011.

9. Loeb GB KA, Canner D, Hiatt JB, Shendure J, Darnell RB, Leslie CS, Rudensky AY: **Transcriptome-wide miR-155 binding map reveals widespread noncanonical microRNA targeting.** Gene Expression Omnibus: https://www.ncbi.nlm.nih.gov/geo/query/acc.cgi?acc=GSE41288; 2012.

10. van Dongen S, Abreu-Goodger C, Enright AJ: **Detecting microRNA binding and siRNA off-target effects from expression data.** *Nat Methods* 2008, **5:**1023-1025.

11. Wong JJ, Au AY, Ritchie W, Rasko JE: **Intron retention in mRNA: No longer nonsense: Known and putative roles of intron retention in normal and disease biology.** *Bioessays* 2015.

12. Wong JJ, Ritchie W, Ebner OA, Selbach M, Wong JW, Huang Y, Gao D, Pinello N, Gonzalez M, Baidya K, et al: **Orchestrated intron retention regulates normal granulocyte differentiation.** *Cell* 2013, **154:**583-595.

13. Theilgaard-Monch K, Jacobsen LC, Borup R, Rasmussen T, Bjerregaard MD, Nielsen FC, Cowland JB, Borregaard N: **The transcriptional program of terminal granulocytic differentiation.** *Blood* 2005, **105:**1785-1796.
