# Supplementary figures and images for: Intron retention enhances gene regulatory complexity in vertebrates
Source: Genome Biol. 2017 Nov 16;18:216. doi: 10.1186/s13059-017-1339-3 (PMC5688624; doi:10.1186/s13059-017-1339-3)

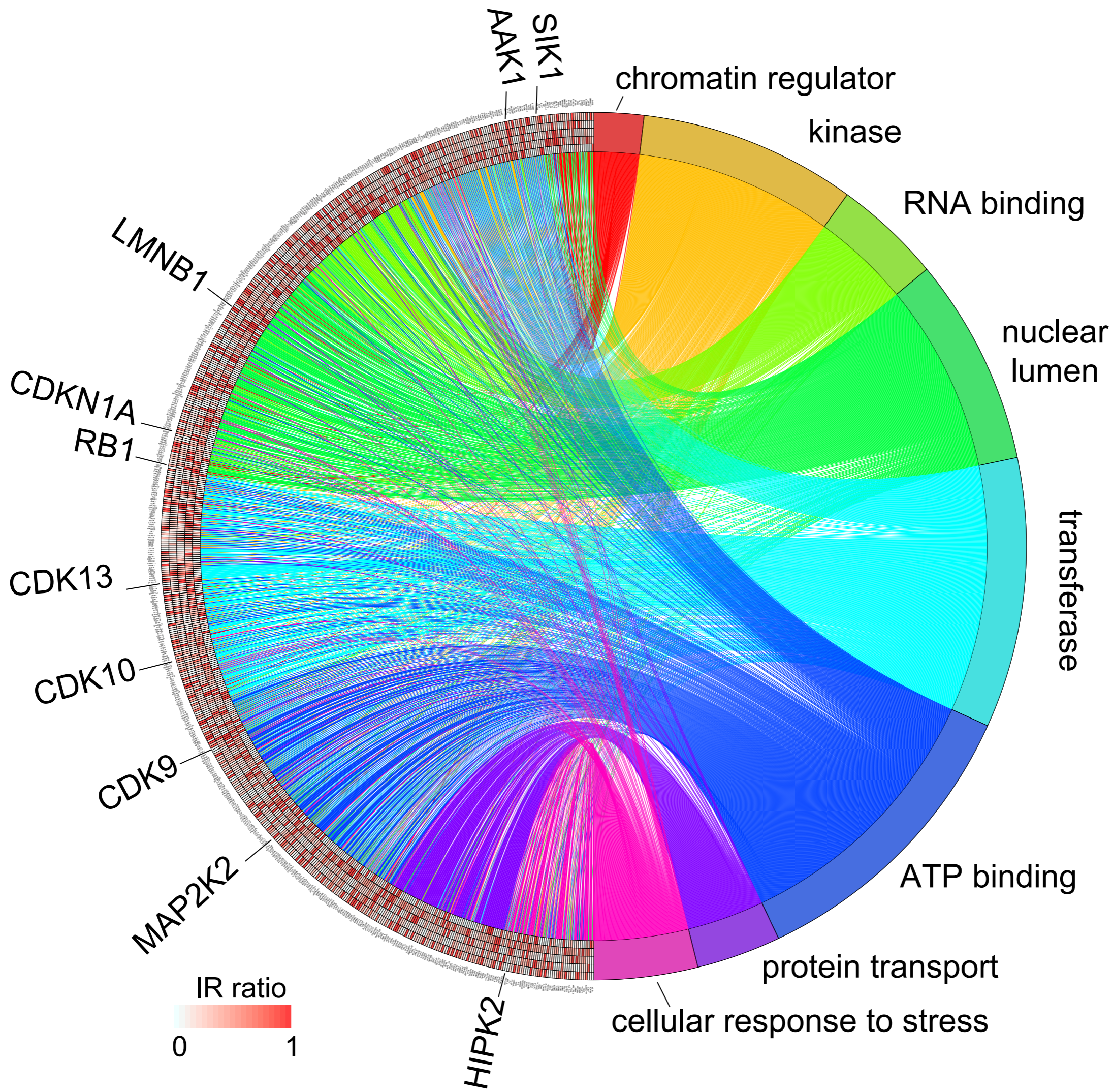

Supplement: Supplementary file 5 — Scalable version of Fig. 1c. (PDF 8057 kb) [file 13059_2017_1339_MOESM5_ESM.pdf]
